# Not so smart? “Smart” drugs increase the level but decrease the quality of cognitive effort

**DOI:** 10.1126/sciadv.add4165

**Published:** 2023-06-14

**Authors:** Elizabeth Bowman, David Coghill, Carsten Murawski, Peter Bossaerts

**Affiliations:** ^1^ Centre for Brain, Mind, and Markets, University of Melbourne, Parkville, Victoria 3010, Australia.; ^2^ Departments of Paediatrics and Psychiatry, University of Melbourne, Parkville, Victoria 3010, Australia.; ^3^ Faculty of Economics, University of Cambridge, Cambridge CB3 9DD, UK.

## Abstract

The efficacy of pharmaceutical cognitive enhancers in everyday complex tasks remains to be established. Using the knapsack optimization problem as a stylized representation of difficulty in tasks encountered in daily life, we discover that methylphenidate, dextroamphetamine, and modafinil cause knapsack value attained in the task to diminish significantly compared to placebo, even if the chance of finding the optimal solution (~50%) is not reduced significantly. Effort (decision time and number of steps taken to find a solution) increases significantly, but productivity (quality of effort) decreases significantly. At the same time, productivity differences across participants decrease, even reverse, to the extent that above-average performers end up below average and vice versa. The latter can be attributed to increased randomness of solution strategies. Our findings suggest that “smart drugs” increase motivation, but a reduction in quality of effort, crucial to solve complex problems, annuls this effect.

## Teaser

“Smart drugs” increase effort but quality deteriorates, particularly affecting productivity of above-average performers.

## INTRODUCTION

Stimulant prescription-only drugs are increasingly used by employees and students as “smart drugs,” to enhance workplace or academic productivity (*1*–*4*). However, even if there is a subjective belief that these drugs are effective as cognitive enhancers in healthy individuals, evidence to support this assumption is, at best, ambiguous (*5*). While improved cognitive capacities such as working memory have been shown, these effects appear to be more evident in clinical samples than the general population (*6*–*9*), a finding that may be explained by ceiling effects. Most puzzling is that, even in clinical populations, mitigation of cognitive deficits has only mild benefits for functioning, for example, at school or in the workplace (*4*), which could be related to the finding in clinical trials that impact on executive function is smaller and/or dose related (*10*, *11*). Thus, a meaningful impact of such drugs on real-world function is yet to be convincingly established.

**Fig. 1.**
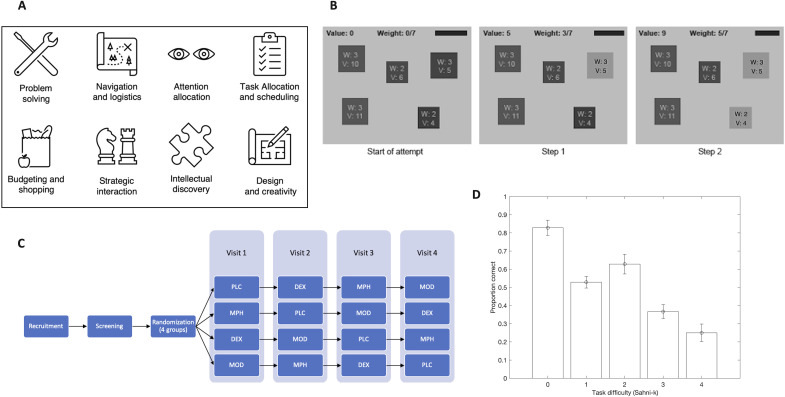
Task relevance, experiment design, and overall participant performance. (**A**) Computationally difficult tasks are ubiquitous in everyday life. (**B**) Task interface with example instance (grayscale version; original in color). Items become highlighted as they are selected. (**C**) Timeline of experiment and Latin square randomization across four experimental sessions. (**D**) Proportion of correct solutions submitted, stratified by task difficulty (Sahni-k index, from low 0 to high 4); circle: estimate of proportion; bars, ±2 SE.

It is often underappreciated just how difficult the tasks that humans encounter in modern life are. At an abstract level, many everyday tasks (Fig. 1A) belong to a mathematical class of problems that is considered “hard,” a level of difficulty not captured by cognitive tasks used in past stimulant studies [technically, these problems are in the complexity class NP (nondeterministic polynomial) hard] (*12*). Typically, they are combinatorial tasks that require systematic approaches (“algorithms”) for optimal outcomes. In the worst case, the number of computations required increases with the size of the problem instance (number of ways to repair a product, number of items available for purchase, number of stops to be made on a delivery trip, etc.) such that it quickly outgrows cognitive capacities. Approximating solutions is not a panacea, as this can be as hard as finding the solution itself (*13*).

We report results from an experiment designed to determine whether and how three popular smart drugs work using a task that encapsulates the difficulty of real-life daily tasks: the 0-1 knapsack optimization problem (“knapsack task”). Participants were asked to choose, from a set of *N* items of differing weights and values, the subset that fits a knapsack of specified capacity (weight constraint) while maximizing total knapsack value. We presented instances of the knapsack task by means of a user interface with less taxation of working memory and arithmetic compared to purely numerical interfaces or interfaces that do not track values and weights of current choices (Fig. 1B). Besides placebo (PLC), the three drugs administered were methylphenidate (MPH), modafinil (MOD), and dextroamphetamine (DEX).

Armed with putative actions of these drugs, we hoped to shed light on why our results emerged. The drugs MPH and DEX are primarily indirect catecholaminergic agonists: They enhance dopaminergic activity in cortical and subcortical areas while also promoting norepinephrine activity (*14*). MPH is an inhibitor of the dopamine transporter; it also weakly inhibits the norepinephrine transporter. DEX shares this mechanism while also augmenting dopamine release into the synapse through interactions with a vesicular monoamine transporter (*15*). The effects of MOD on cortical and subcortical catecholamines have proved far more challenging to uncover: It has an inhibitory effect on dopamine transportation (*16*, *17*) while influencing norepinephrine transportation as well (*18*), but it also increases glutamate in the thalamus and hippocampus and reduces γ-aminobutyric acid in the cortex and hypothalamus (*19*, *20*). We expected that because of increased dopamine, the drugs induced would increase motivation and, in conjunction with a concurrent increase in norepinephrine, cause an increase in effort expended on the task, which in turn would lead to higher performance.

Forty participants, aged between 18 and 35 years, participated in a randomized double-blinded, PLC-controlled single-dose trial of standard adult doses of the three drugs (30 mg of MPH, 15 mg of DEX, and 200 mg of MOD) and PLC, administered before being asked to solve eight instances of the knapsack task. Doses are at the high end of those administered in clinical practice, reflecting typical doses in nonmedical settings, where use tends to be occasional rather than chronic. Ethics approval was obtained from the University of Melbourne (HREC 1749142; registered as clinical trial PECO: ACTRN12617001544369, U1111-1204-3404). Participants attempted each instance twice. A time limit of 4 min was imposed, which was binding in only ~1% of valid responses. The four experimental sessions were at least 1 week apart from each other. Participants were randomly assigned to conditions using a Latin square design (Fig. 1C). To gauge the comparability of our results to those from prior experiments, participants were also asked to complete four tasks from the CANTAB cognitive battery (the simple and five-choice reaction time task, the stockings of Cambridge Task, the spatial working memory task, and the stop-signal task) (*21*).

Given the well-documented erratic nature of the effects of the drugs on baseline cognitive functions (*10*, *11*) and the lack of understanding of how baseline cognitive functions translate into success on complex combinatorial tasks such as the knapsack task, we refrain from formulating hypotheses about the results to be expected. Instead, we adhered strictly to stringent statistical model selection protocol, using Akaike and Bayesian information criteria, to select the best-fitting models. We then performed statistical tests only on those models (see Materials and Methods).

## RESULTS

### Performance decreases with instance-specific metrics of difficulty

Participants solved 50.3% of instances correctly (SEM = 0.9%). Instances differed in difficulty. To characterize the latter, we used a metric, Sahni-k, that has successfully predicted performance of human participants in the knapsack task in earlier experiments (*22*–*24*). According to this metric, an instance is “easy” (Sahni-k = 0) if it can be solved using the greedy algorithm, which is to fill the knapsack with items in decreasing order of the ratio of value/weight until the capacity limit is reached. If *n* items must be in the knapsack before the greedy algorithm can be used to produce the solution, then Sahni-k = *n*. Difficulty thus increases with Sahni-k. In our experiment, Sahni-k varied across instances, from 0 to 4 (see Materials and Methods). Confirming findings of earlier experiments (*22*–*24*), we observed a significant decrease in performance (proportion of correct attempts) as Sahni-k increased (slope = −0.56, *P* < 0.0001; Fig. 1D and table S1).

We used two additional metrics of difficulty: (i) DP complexity, a difficulty metric derived from the dynamic programming algorithm used to solve knapsack problems (*25*), and (ii) props, the number of propagations, and hence, the time it takes MiniZinc, a widely used general-purpose solver for hard computational problems (*26*). Human performance often shows little simple correlation with these difficulty metrics (figs. S1 and S2), but they are included in the analysis because they explain part of the performance variance left unexplained by Sahni-k. The difficulty metrics are positively but imperfectly correlated (see Materials and Methods).

### Drugs did not affect the chance of finding the correct solution

We first examined the impact of the drugs on a participant’s ability to solve an instance. To this end, we estimated a logistic model relating performance to instance difficulty and drug condition, accounting for possible interactions and participant-specific random effects. We always considered several different model specifications and report the one with the best goodness of fit (see Materials and Methods for details). The best-fitting model was one that pooled the active drug conditions and where random effects on the intercept term at the individual level were accounted for, and two difficulty metrics were included as explanatory variables for performance, Sahni-k and DP complexity. There was no significant effect of drug on performance (slope = −0.16, *P =* 0.11; see table S1).

### Drugs decreased value attained

**Fig. 2.**
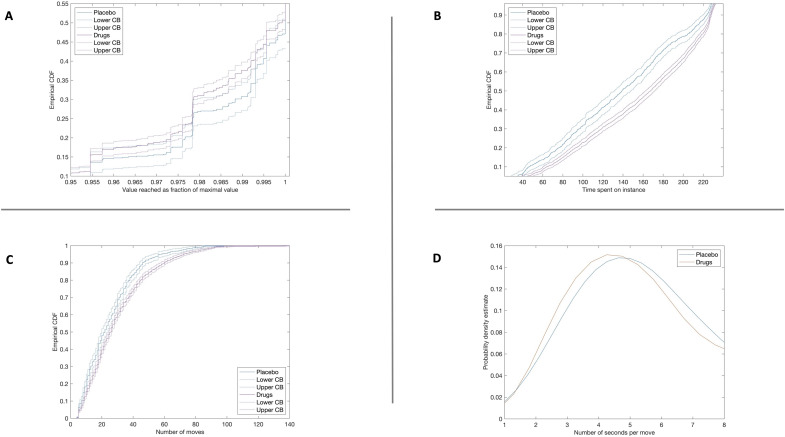
Performance, effort, and speed. (**A** to **C**) Empirical cumulative distribution function under PLC (blue) and drugs (red) and pointwise 95% confidence bounds (CB; based on Greenwood’s formula). (A) Knapsack value reached as a fraction of maximal value. PLC first-order stochastically dominates drugs, implying that the chance that participants reach any value is uniformly lower under drugs than under PLC. (B) Effort is equal to the time spent until submission of solution. Drugs first-order stochastically dominates PLC, implying that the chance of spending any amount of time is uniformly higher under drugs than under PLC. (C) Effort is equal to the number of moves of items in/out of knapsack until submission of solution; drugs first-order stochastically dominates PLC, implying that the chance of executing any number of moves is uniformly higher under drugs than under PLC. (**D**) Probability density estimates of speed under PLC (blue) and drugs (red), where speed is equal to the number of seconds per move. Because the density under drugs is shifted to the left of that under PLC, speed tends to be higher under drugs than under PLC.

Next, we investigated the effect of the drugs on the value attained in an attempt. We found that drugs had a negative effect on value (slope = −0.003, *P* = 0.02; table S2), that is, participants tended to achieve a lower value in the instances in the drug conditions. A plot of the distribution of attained values in the drug conditions against that under PLC shows that the negative effect extends to the entire distribution: The chance that success is below any given level is greater under drugs than under PLC (pointwise 95% confidence intervals mostly fail to intersect; Fig. 2A).

### Drugs increased time spent

We then turned to effort expended. For this, we examined the time participants spent on an instance before submitting their suggested solution. Participants spent substantially more time on an instance in the drug conditions [slope(DEX) = 18.8; slope(MPH) = 29.1; both *P* < 0.0001; slope(MOD) = 9.1, *P* = 0.10; table S3]. Inspection of the distribution function of time spent reveals a sizeable and significant move of the distribution under drug conditions to the left relative to that under PLC (pointwise 95% confidence intervals fail to intersect except in the tails; Fig. 2B). The increase in time spent under MPH is equivalent to an increase in difficulty (Sahni-k) of more than 4 points. That is, participants spent almost as much time on the easiest instances under MPH as on the hardest instances under PLC, without any corresponding improvement in performance.

### Drugs increased number of moves

Another index of effort is the number of moves of items in and out of the suggested solution undertaken while attempting to solve an instance (indicated by clicking on the item icon in the user interface; see Fig. 1B). Drugs increase the number of item moves: DEX, 7.2 moves (*P* < 0.0001); MPH, 6.1 moves (*P* < 0.0001); and MOD, 1.9 moves (*P* > 0.1; table S3). The distribution of moves shifts leftward under drugs (Fig. 2C), analogous to the shift observed in relation to time spent (Fig. 2B). The size of the effect on moves of DEX and MPH is the same as increasing difficulty (Sahni-k) by more than 2 points. Because both time spent and moves taken increase in the drug conditions, the effect on speed is unclear. Figure 2D shows that the distribution of the number of seconds per move shifted to the left, but regression analysis (table S5) fails to produce significant relations (*P* > 0.05). Thus, if one measures motivation in terms of time spent or number of items moved, drugs clearly enhanced motivation. If, however, motivation is to be captured by speed, the evidence is mixed.

### Drugs significantly decrease quality of effort

**Fig. 3.**
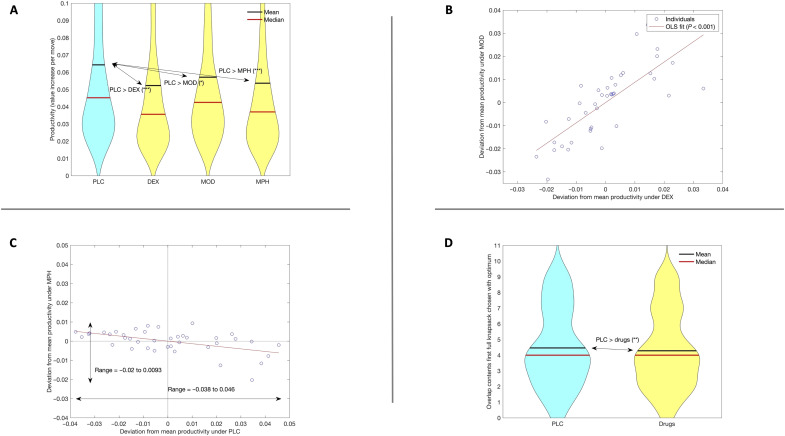
Quality of effort. (**A**) Violin plots of productivity, measured as average increase in value of knapsack per item move in/out of knapsack. Stars indicate significance of differences in means based on a generalized linear model that accounts for confounding factors and participant-specific random effects for average productivity and impact of drugs (table S6); *P < 0.05 and ***P < 0.001. (**B** and **C**) Estimated participant-specific (random) deviations in productivity from mean productivity. Productivity is measured as average increase in value of knapsack per item move; random effects were estimated with a generalized linear model that accounts for confounding factors and participant-specific random effects for average productivity and impact of drugs (table S6). (B) MOD against DEX. The red line shows OLS fit, with significant positive slope (*P* < 0.001). (C) MPH against PLC. The red line shows OLS fit, with significant negative slope (*P* < 0.001). Arrows indicate range of productivity deviations under PLC (horizontal) and MPH (vertical). The range is smaller under MPH than under PLC, implying reversion to the mean. (**D**) Reduction in quality of first full knapsack chosen under drugs (right) relative to PLC (left). Quality is measured as overlap between number of items in chosen knapsack and optimal knapsack. Decrease in mean quality is significant at **P < 0.01, based on a generalized linear model that accounts for effect of instance difficulty and overlap with items in the Greedy solution, as well as participant-specific random effects for average quality (table S7); overlap tends to be lower under drugs than under PLC, implying lower quality of solution search.

We therefore proceeded to study the quality of the moves made by participants. We defined productiveness as the average gain in value per move of attempted knapsacks (as a fraction of optimal value). Figure 3A displays violin plots of productivity for PLC and the three drugs separately. Productivity is uniformly smaller across all drugs (relative to PLC). Regression analysis confirmed a significant and sizable drop in productivity with drugs (all *P* < 0.001; see table S6) with an average decrease in productivity equivalent to increasing task difficulty by 1.5 (Sahni-k) points.

### Drugs cause quality of effort reversals

The mean effect of drugs on productivity masks substantial heterogeneity across participants. Investigation of deviations in individual productivity from the mean under PLC versus under drugs revealed a significant tightening: The range of estimated deviations was reduced by more than half. For MPH, the range dropped from [−0.038, 0.0046] to [−0.02, 0.0092] (see Fig. 3B). A Wilcoxon signed rank test confirmed that individual productivity deviations were stochastically smaller under MPH than under PLC (*P* < 0.0001). This result must not be interpreted as regression to the mean (*27*), as temporal participant assignment to MPH and PLC was random. An analogous statistically significant stochastic reduction was measured for MOD relative to PLC (*P* = 0.02; fig. S4) and for DEX relative to PLC (*P* = 0.002; fig. S5).

Significant negative correlation between productivity under MPH and under PLC emerged [slope of the Ordinary Least Squares (OLS)] fit = −0.13, *P* < 0.001 based on *z*-statistic computed from Maximum Likelihood Estimation (MLE) estimates of correlation of estimated random effects as reported in table S6, correlation is equal to −0.43; Fig. 3B). We thus observed a disturbing performance reversal. Participants who were above the mean under PLC tended to fall below the mean under MPH. Likewise, significant reversals emerged under MOD (correlation of −0.55, *P* < 0.001; fig. S4 and table S6) and under DEX (correlation of −0.21, *P* = 0.01; fig. S5 and table S6).

Across drugs, strong correlation in individual participant deviations in individual productivity from the mean effects across drug conditions emerged (table S6). The correlation was as high as 0.70 for MOD and DEX (the slope of the OLS line, close to 45°, is highly significant: *P* < 0.001; Fig. 3C). Although DEX and MPH are thought to affect neurotransmission in analogous ways, we found a strong negative correlation between individual effects under the two drugs [see fig. S6 (OLS slope = −0.29; *P* < 0.0001)].

### Quality of effort decreases because moves become more random

Last, we examined attempts at a finer level of granularity. Prior work has revealed that the performance of an attempt to solve an instance in the knapsack task depends on the quality of the first full knapsack that a participant composes (*23*). Here, we define quality as the number of items common to the first full knapsack and the optimal knapsack. The quality of the first knapsack was lower in the drug conditions compared to PLC (slope = −0.176, *P* = 0.003; table S8). The mean overlap is significantly lower under drugs than under PLC (Fig. 3D).

The first full knapsack overlaps more with the optimal one if there is more commonality between the solution from the greedy algorithm and the optimal solution, and this correlation increases with instance difficulty (Sahni-k; table S7). This is consistent with earlier findings that the first full knapsack tends to be obtained using the greedy algorithm (*23*). Evidently, drugs tend to make the first full knapsack more random. This, together with the finding that exploration (number of moves) increases, suggests that participants’ approach to solving a hard problem such as the knapsack task becomes less systematic under drugs; in other words, while drugs increase persistence, they appear to reduce the quality of effort.

### Scores on CANTAB tasks do not predict drug effects

We found significant correlation between scores on only two CANTAB tasks (working memory task: *P* < 0.001; simple reaction time task: *P* < 0.01) and performance in the knapsack task (performance was assessed on the basis of whether the submitted solution was correct; see figs. S7 and S8). However, there was no significant interaction with drugs, in that scores on the CANTAB tasks did not predict drug effects in the knapsack task (*P* > 0.10; examples: figs. S9 to S12). Likewise, we were not able to predict individual drug effects in the knapsack task from drug effects on individual scores in the CANTAB tasks (*P* > 0.10; examples: figs. S13 to S16).

## DISCUSSION

While drug treatments did not cause a significant drop in the average chance of finding the solution to the knapsack problem instances, they did lead to a significant overall drop in value attained. Whether defined as time spent or number of moves (of items in/out of the knapsack), effort increased significantly on average. Because both aspects of effort increased, the effect on speed (number of seconds per move) became ambiguous.

The most notable aspect of our findings concerns heterogeneity in quality of effort, however. Effort quality was defined as the average increase in knapsack value per move. We found a significant stochastic reduction in the magnitudes of individual deviations from mean effort quality under each drug, compared to PLC. That is, heterogeneity in effort quality under drugs stochastically dominated that under PLC.

In addition, significant negative correlation between individual deviations from average effort quality between each drug and PLC emerged. This means that, if an individual exhibited above-average increase in knapsack value per move under PLC, she tended to be below average under MPH, DEX, and MOD. Conversely, if an individual performed below average under PLC, the quality of effort was above average under MPH, DEX, and MOD.

We found that this reversal in effort quality emerged because participants became more erratic in their choices when under drugs: The first full knapsack that they considered was more random than under PLC. This disproportionally affected above-average participants; those that performed below average under PLC did increase their effort quality merely because they spent more effort (spent more time).

Our task was computationally hard, and hence, optimal choices require systematic thought. Random exploration is not effective in this task, in contrast with probabilistic tasks, where strategies such as epsilon-greedy or softmax can be optimal (*28*). Because quality of choice is secondary in probabilistic tasks, it is expected that for these, drugs such as MPH or MOD have been observed to improve performance, albeit mildly (*29*–*34*).

Good effort allocation is primordial for the knapsack task. It has been argued that dopamine and norepinephrine, two neuromodulators targeted by the drugs administered in this study, regulate the trade-off between reward and effort cost (*35*) and that this trade-off is governed by the overarching goal of maximizing the expected value of control; the latter steers not only the quantity of effort but also the type of effort chosen (referred to as efficacy). Evidently, this theory elucidates the workings of the drugs that we administered: They boost subjective reward while reducing perceived effort, but they have a detrimental effect on efficacy.

The drugs that we administered are known to reduce performance of healthy participants in some of the CANTAB tasks that we included in our experiment (*6*–*9*). We confirmed these effects and extended them to the knapsack task. However, we failed to predict individual drug effects in the knapsack task from scores on the CANTAB tasks or from drug effects in the CANTAB tasks.

When compared to recorded effects on baseline cognition (CANTAB tasks) in patients with attention deficit hyperactivity disorder (ADHD) (*8*, *10*, *11*), there appears to be overlap: Evidence of effects is scattered, and if they emerge, then effects are characterized by considerable heterogeneity. Hence, the evidence from healthy participants appears to be an extension of that of the clinical population, so that ADHD may not be a categorical disorder but instead better described as a dimensional disorder (*36*, *37*).

Because the knapsack task encapsulates difficulty encountered in everyday problem-solving, our paradigm could help shed light on how medications such as MPH improve the day-to-day functioning of patients suffering from, e.g., ADHD. In addition, the knapsack task facilitates the much-needed comparison across clinical and subclinical populations (*36*). Last, for subclinical populations, our paradigm provides a convenient framework with which to eventually discover the genuinely smart drugs, i.e., the drugs that not only increase effort but also enhance quality of effort.

## MATERIALS AND METHODS

### Experimental protocol

Forty healthy male (*n* = 17) and female (*n* = 23) volunteers between the ages of 18 and 35 (mean, 24.5 years) were recruited from campus advertisements. All volunteers were screened by a clinician via semistructured interview and examination before enrollment in the study. Study exclusion criteria included history of psychiatric or neurological illness including epilepsy or head injury, previous use of psychotropic medication, history of substantial drug use, heart conditions (including high blood pressure, defined as above 140 mm/Hg systolic and/or 90 mm/Hg diastolic pressure as measured at the initial assessment session), pregnancy, or glaucoma. A brief cardiac examination was performed, and any family history of sudden death of a first-degree relative through cardiac or unknown causes before the age of 50 years old also excluded the participant. Participants were asked to refrain from any alcohol and caffeine from midnight the night before each testing session.

Participants were required to attend four testing sessions, each session spaced at least 7 days from the previous session. At each session, participants received one of either 200 mg of MOD, 30 mg of MPH, 15 mg of DEX, or microcrystalline cellulose (Avicel) PLC. All medications were dispensed as identical white capsules in double-blinded packaging. Participants were randomly allocated into four groups, each group receiving a different sequence of medications and PLC across sessions according to a counterbalanced Latin square design (see Fig. 1B). Randomization sequences were generated by the Melbourne Clinical Trials Centre (Melbourne Children’s Campus).

Participants arrived at the testing venue in the morning and had their blood pressure measured after at least 5 min of sitting quietly. The capsule for the session was given with a glass of water, and a 90-min waiting period commenced. Participants were encouraged to bring study or quiet reading to do during this period. After 90 min, participants’ blood pressure was measured, and they then completed the complex optimization and cognitive tasks. Upon completion of all the tasks, participants’ blood pressure was measured one final time, and participants were then free to go. The experiment was registered as a clinical trial (PECO: ACTRN12617001544369, U1111-1204-3404). Ethics approval was obtained from the University of Melbourne (HREC1749142).

### The knapsack task

The knapsack optimization problem (“knapsack task”) is a combinatorial optimization task, where the participant is presented with a number of items, each item having an associated weight and value. The goal is to find the combination of items that maximizes the combined value of the selected items, while the combined weight of the items remains beneath a given weight limit. The knapsack task is in the class of NP-time hard problems.

Participants were presented with eight unique instances of the knapsack task, with each instance containing 10 or 12 different items and a different weight limit. The task was presented via laptop, and participants clicked on items to select or deselect them from their solution. The problem’s weight limit and the cumulative weight and value of the selected items were displayed at the top of the screen. Participants were prevented from selecting items that would exceed the weight limit. A 4-min limit was imposed on each presentation of the problem, and participants could submit their solution at any time during those 4 min by pressing the space bar. Participants were not informed whether their solution was optimal or not, and each instance was presented twice. Each selection or deselection of an item before submission, as well as the timing of each choice, is recorded for later analysis.

The same eight instances were used as those reported in (*23*). Details of the instances, including solutions, can be found there. Table 1 lists the instances along with metrics of difficulty used here. Instances are numbered as in the article.

**Table 1.** Difficulty of instances used in the knapsack task.

Instance (#items)	1 (10)	2 (10)	3 (12)	4 (10)	5 (12)	6 (12)	7 (12)	8 (10)
**Sahni-k**	1	3	2	0	1	1	4	3
**DC complexity**	109	100	117	105	46	143	124	80
**MiniZinc #props**	3577	1117	25,961	4133	6278	19,417	16,498	9155

Difficulty metrics are positively but far from perfectly correlated. Table 2 displays the correlation matrix.

**Table 2.** Correlations between difficulty metrics. SE = 0.20 (based on Fisher’s transformation).

	DC complexity	MiniZinc #props
Sahni-k	0.08	0.20
DC complexity		0.52

### CANTAB tasks

#### Simple and five-choice reaction time task

The reaction time tasks assess the participants’ response speed to a visual cue in either a predictable location (the simple variant) or in one of five locations (the five-choice variant). The mean duration between releasing the response button and touching the target button, calculated across all correct trials, is the major outcome of interest.

#### Stockings of Cambridge

The stockings of Cambridge task examines spatial planning and, to a lesser extent, spatial working memory. The participant is required match a sequential pattern of balls while following rules regarding the permitted movement of the balls in space. The task difficulty varies by the minimum number of moves required to match the given pattern and ranges from two to five moves. The major outcome of interest is the number of patterns matched in the minimum moves, calculated across all correct trials. The change in number of correct trials with increasing difficulty can also be examined. Note that on one occasion, the app-based task failed to run, resulting in no data for that task for that session.

#### Spatial working memory

The spatial working memory task is a test of the participant’s ability to retain spatial information in working memory. The participant is required to collect tokens hidden in a randomly placed array of boxes, where a found token will never reappear inside the same box. Task difficulty is increased by increasing the number of tokens and boxes, starting with 4, and progressing through 6, 8, and 12 box arrays. Performance is most often calculated as a “strategy score,” that is, the number of times their search for the token started from the same box, implying that a specific spatial strategy is used. Between error and within error counts are also often examined, being the number of times a box in which a token has previously been found is revisited, the number of times a participant revisits a box already shown to be empty.

#### Stop signal task

The stop signal task is a test of response inhibition, generating an estimate of stop signal reaction time using staircase functions. The participant presses a left-hand button when a cue arrow indicates left and a right-hand button when the cue indicates right, except for when a tone is heard. If a tone is heard, the participant should refrain from pressing the button. The timing of the tone in relation to the cue is adjusted throughout the trial, depending on performance, until the participant is able to stop in only approximately 50% of trials. This duration between cue and tone is the major measure of interest.

### Statistical analysis

Formal statistical tests of drug effects, both at the population level, and if deemed appropriate, at the individual level, are based on random-effects generalized linear modeling using the MATLAB function glmfit in release 2022b (The MathWorks Inc., MA, USA). Absent specific hypotheses, model specification, including whether (individual) random effects had to be included and at what level (per drug), or for all drug treatments combined, was based on strict adherence to model selection using the Akaike and Bayesian information criteria.

MATLAB code that generates the statistics and figures, along with underlying data, can be found in the notebook “figures.mlx” and “SOM.mlx” of the GitHub repository bmmlab/PECO (https://zenodo.org/badge/latestdoi/592775835). The MATLAB code allows the reader to understand exactly the nature of the model estimated. The code also facilitates replication. The combination of code and data allows the reader to replicate all statistical results reported in the article and its Supplementary Materials, as well as generating all tables and figures. Tests of stochastic dominance of individual random effects under drugs versus under PLC were based on the Wilcoxon signed rank test of the null that the sizes (squares) of the individual random effects are exchangeable under the treatments.

## Supplementary Materials

### This PDF file includes:

Figs. S1 to S16 Tables S1 to S7 References

## Notes

View/request a protocol for this paper from *Bio-protocol*.
