# Supplementary material for: Not so smart? “Smart” drugs increase the level but decrease the quality of cognitive effort
Source: Sci Adv. 2023 Jun 14;9(24):eadd4165. doi: 10.1126/sciadv.add4165 (PMC10266726; doi:10.1126/sciadv.add4165)
Supplement: Supplementary file 1 — Figs. S1 to S16 Tables S1 to S7 References [file sciadv.add4165_sm.pdf]

Science Advances
AAAS

# Supplementary Materials for

## Not so smart? "Smart" drugs increase the level but decrease the quality of cognitive effort

Elizabeth Bowman *et al.*

Corresponding author: Peter Bossaerts, plb32@cam.ac.uk

*Sci. Adv.* **9**, eadd4165 (2023)
DOI: 10.1126/sciadv.add4165

**This PDF file includes:**

Figs S1 to S16
Tables S1 to S7
References

## Supplementary Materials: Do "Smart" drugs make one smarter?

**Contents**

Supplementary Figs S1 to S16
Supplementary Tables S1 to S7

Supplementary Figures

(Matlab code that generates the figures, along with underlying data, can be found in the notebook "SOM.mlx" at https://zenodo.org/badge/latestdoi/592775835)

Figure S1: Proportion of attempted solutions that were correct, by quintile of difficulty where difficulty is measured in terms of log2(Capacity)*(number of items) ("DC Complexity")

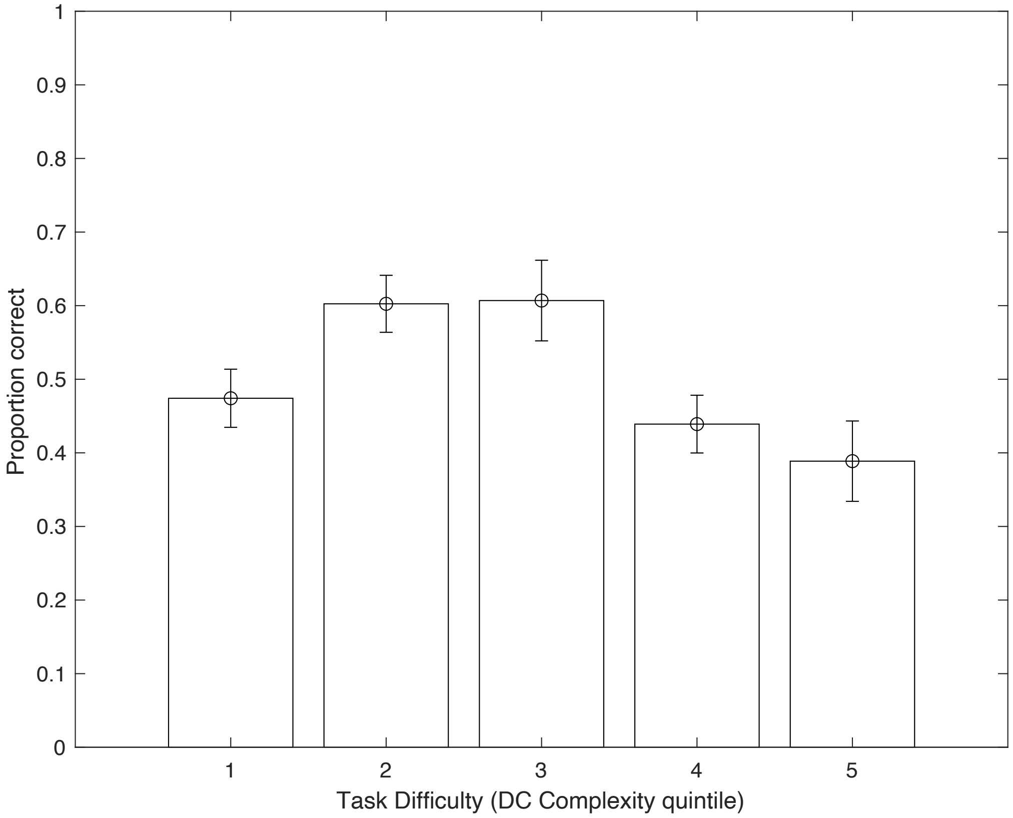

Figure S2: Proportion of attempted solutions that were correct, by quintile of difficulty where difficulty is measured in terms of number of propagations the program MiniZinc needs to find a solution (#Comps)

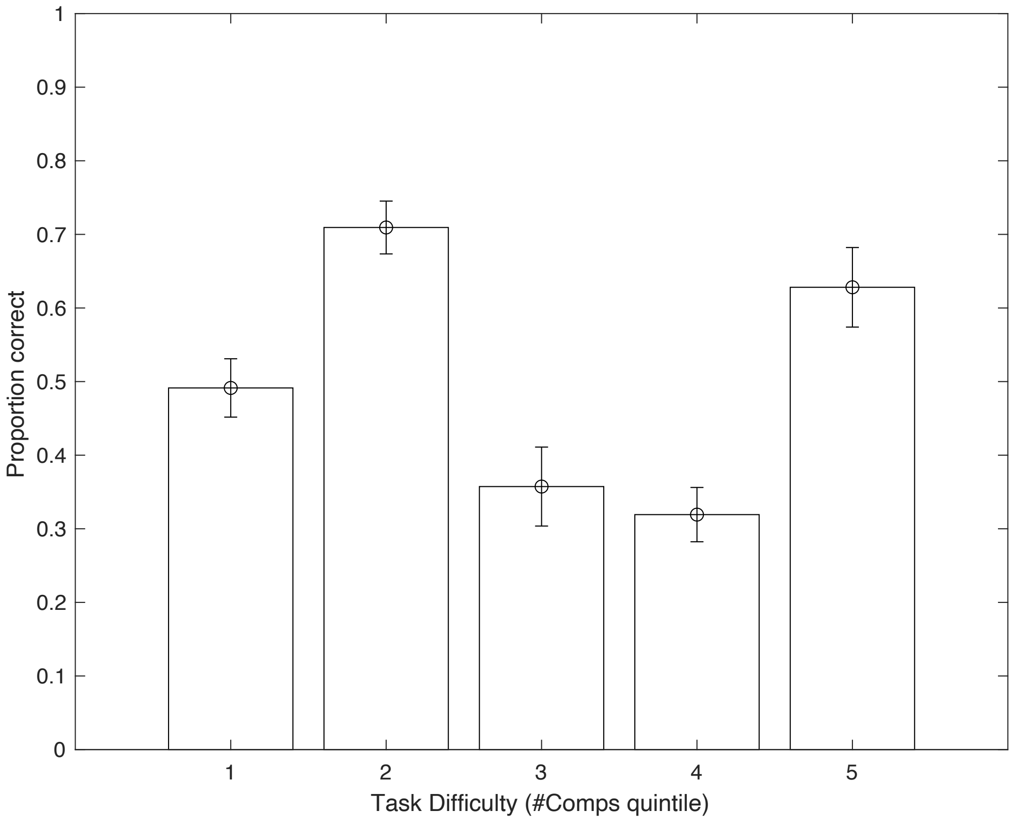

<u>Figure S3</u>: Plot of Participant Random Effects under PLC and under Drugs from GLM Regression of Score on Knapsack Task onto Sahni_k, Complexity and Drugs dummy, with Random Effects for PLC and Drugs

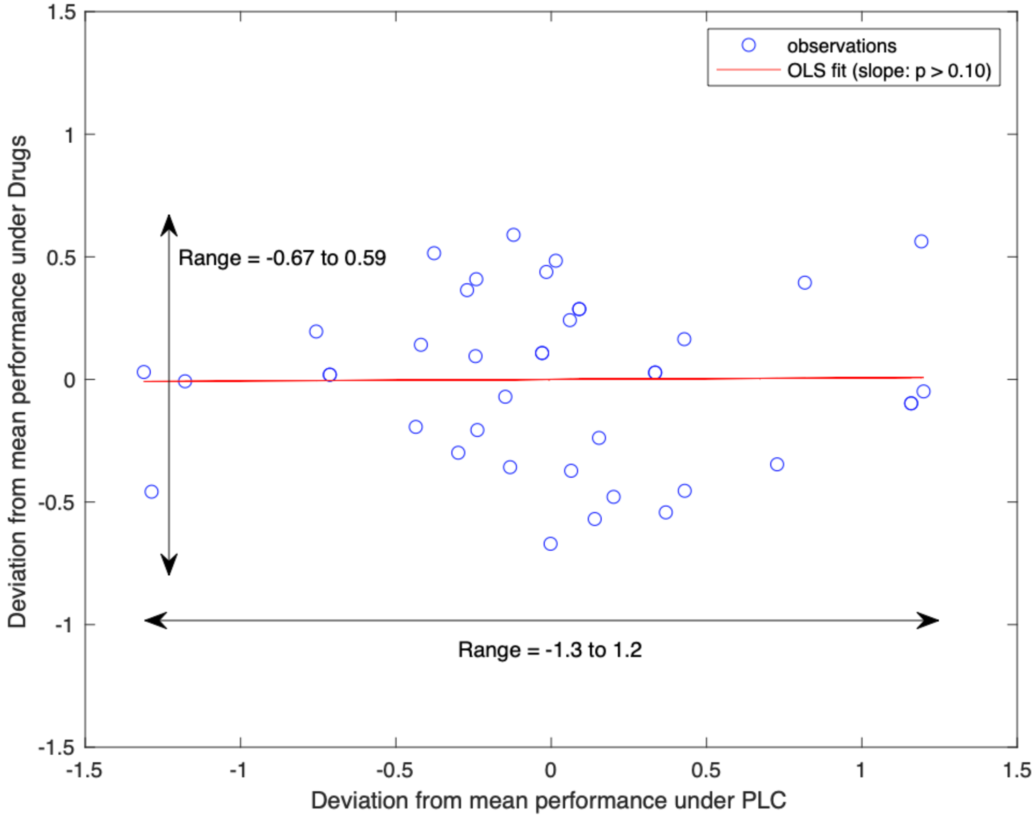


*Note:* Table S1, unlike Figure S3, display regression results without separate random effects under Drugs; with them, the AIC and BIC model selection criteria deteriorate to 11372 and 11413 respectively. A Wilcoxon signed rank test confirmed that individual performance deviations plotted in Figure S3 were exchangeable rather than stochastically smaller under Drugs than under PLC ($p > 0.10$).

Figure S4: Plot of Participant Random Effects of Productivity under MOD vs. under PLC (from regression in Table S6). Wilcoxon signed rank test confirmed that individual productivity deviations were stochastically smaller under MOD than under PLC ($p = 0.02$).

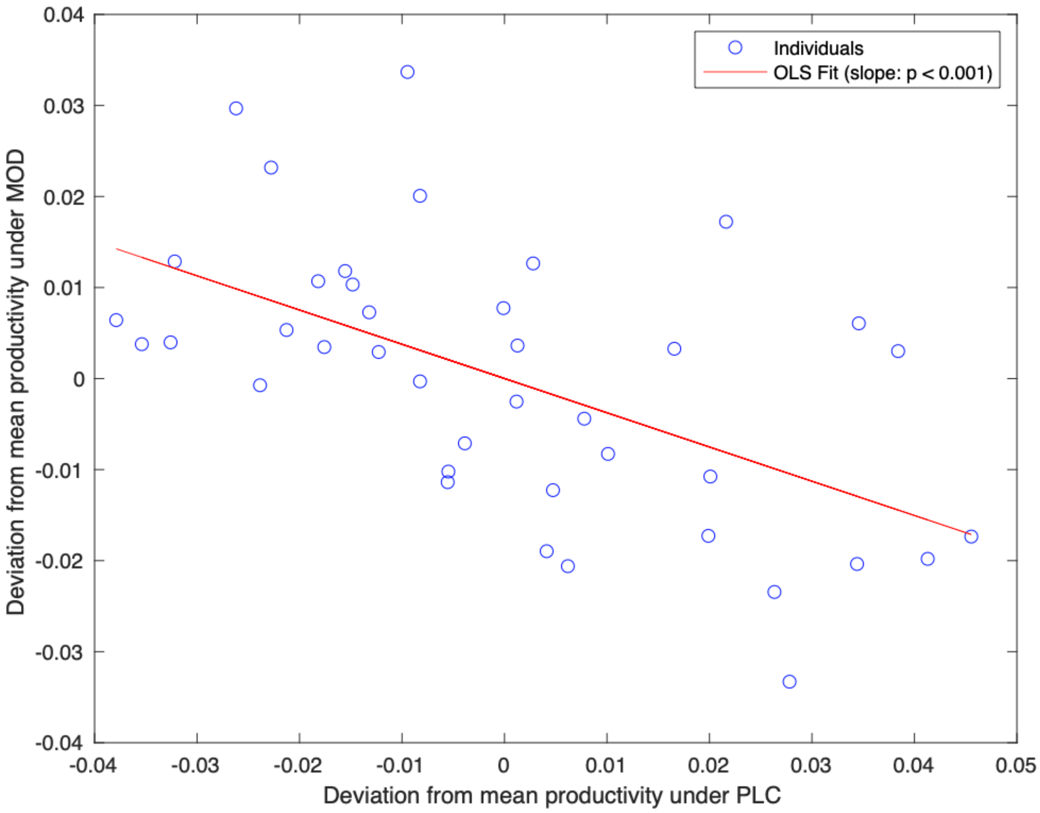

Figure S5: Plot of Participant Random Effects of Productivity under DEX vs. under PLC (from regression in Table S6). Wilcoxon signed rank test confirmed that individual productivity deviations were stochastically smaller under DEX than under PLC ($p = 0.002$).

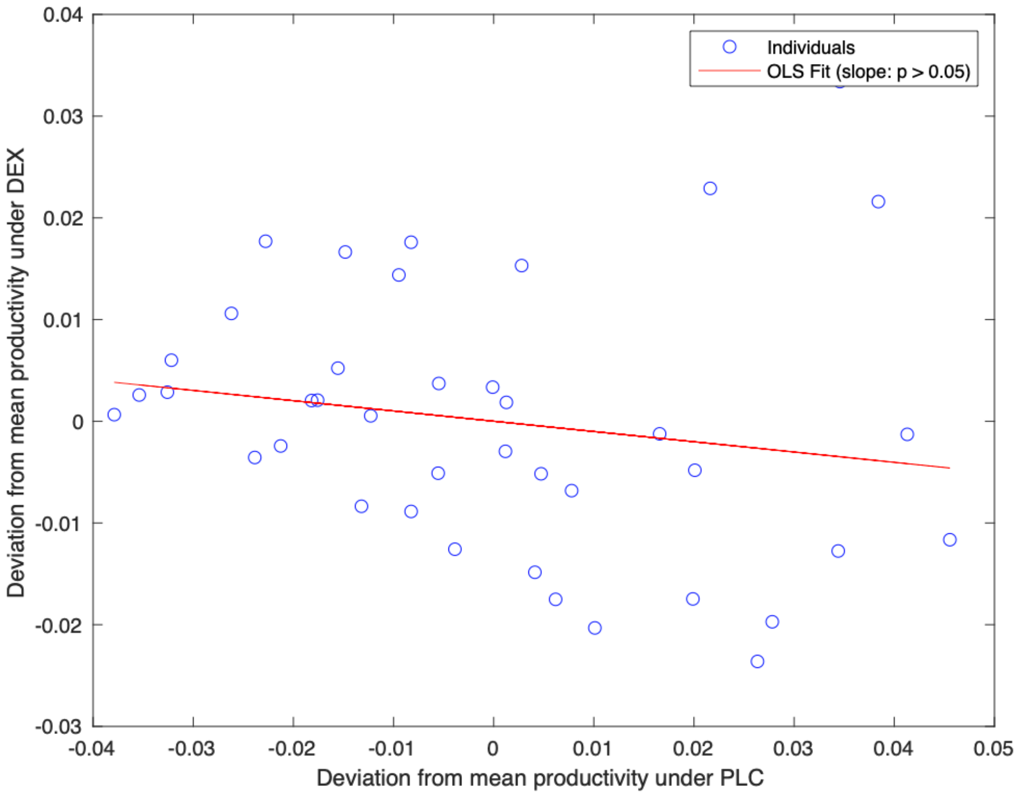

Figure S6: Plot of Participant Random Effects of Productivity under DEX vs. under MPH (from regression in Table S6)

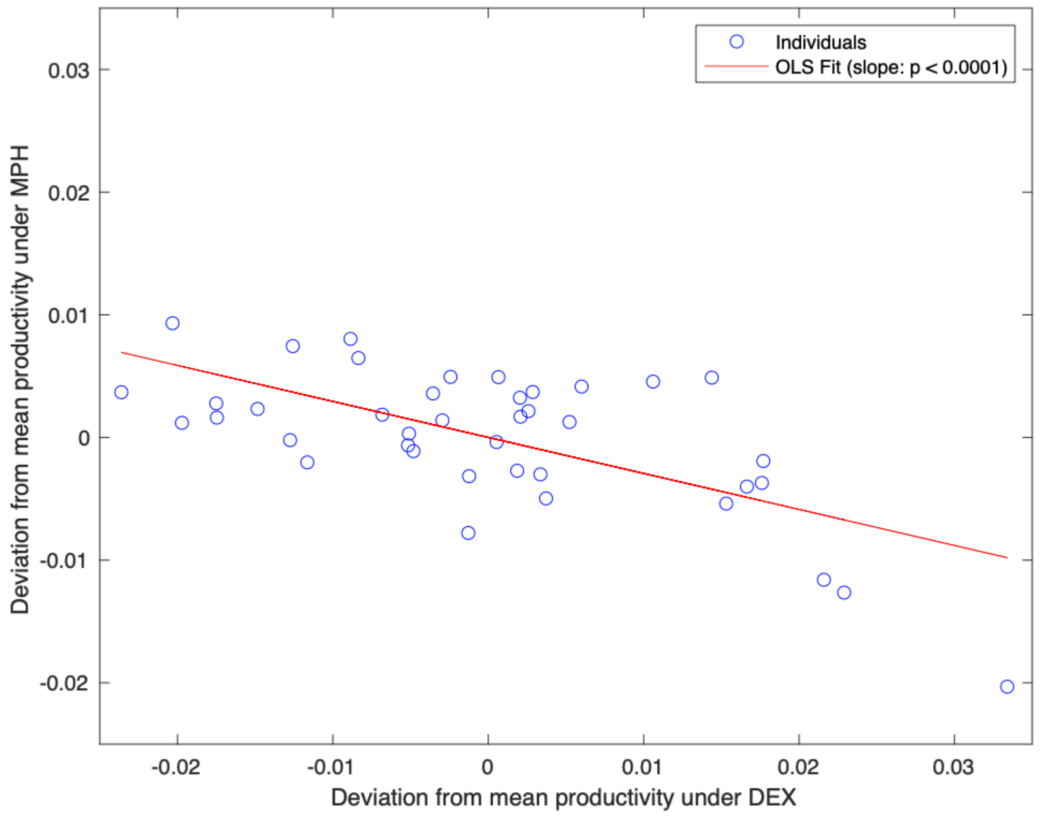

Figure S7: Plot of Participants Random Effects from Table S1 (Explaining Score on Knapsack Task) Against Average Score on CANTAB Spatial Working Memory Task (SWMSX).

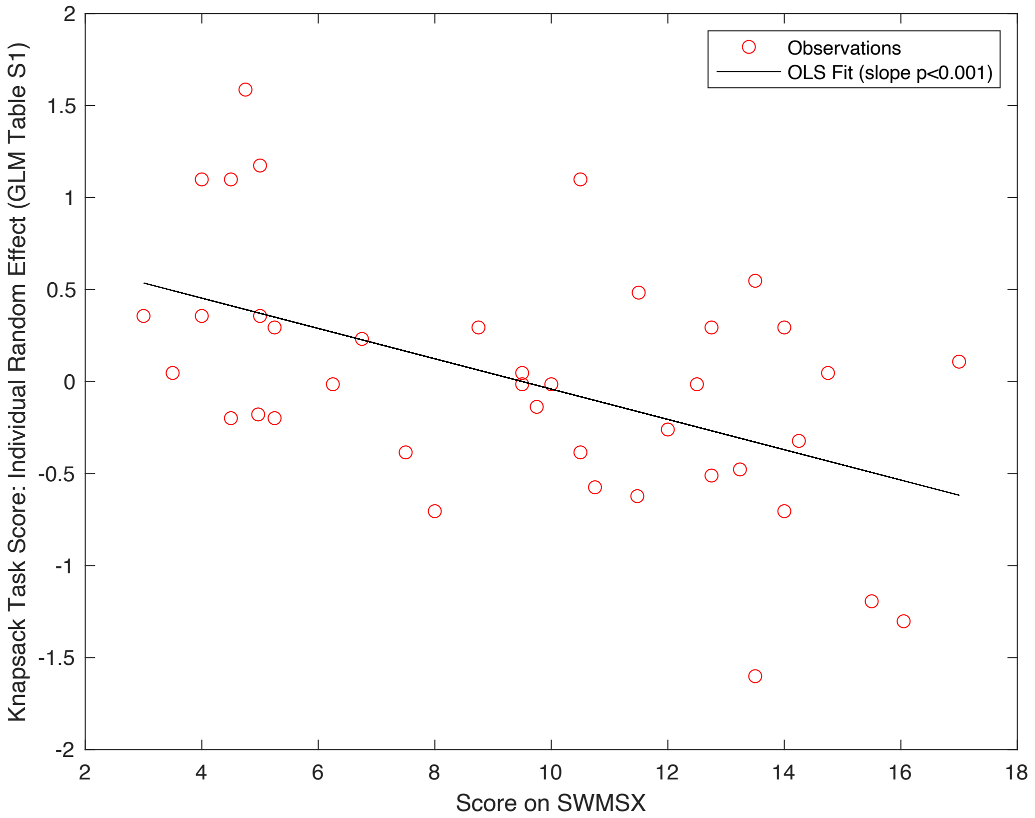

Figure S8: Plot of Participants Random Effects from Table S1 (Explaining Score on Knapsack Task) Against Average Score on CANTAB Simple Reaction Time Task (SRT).

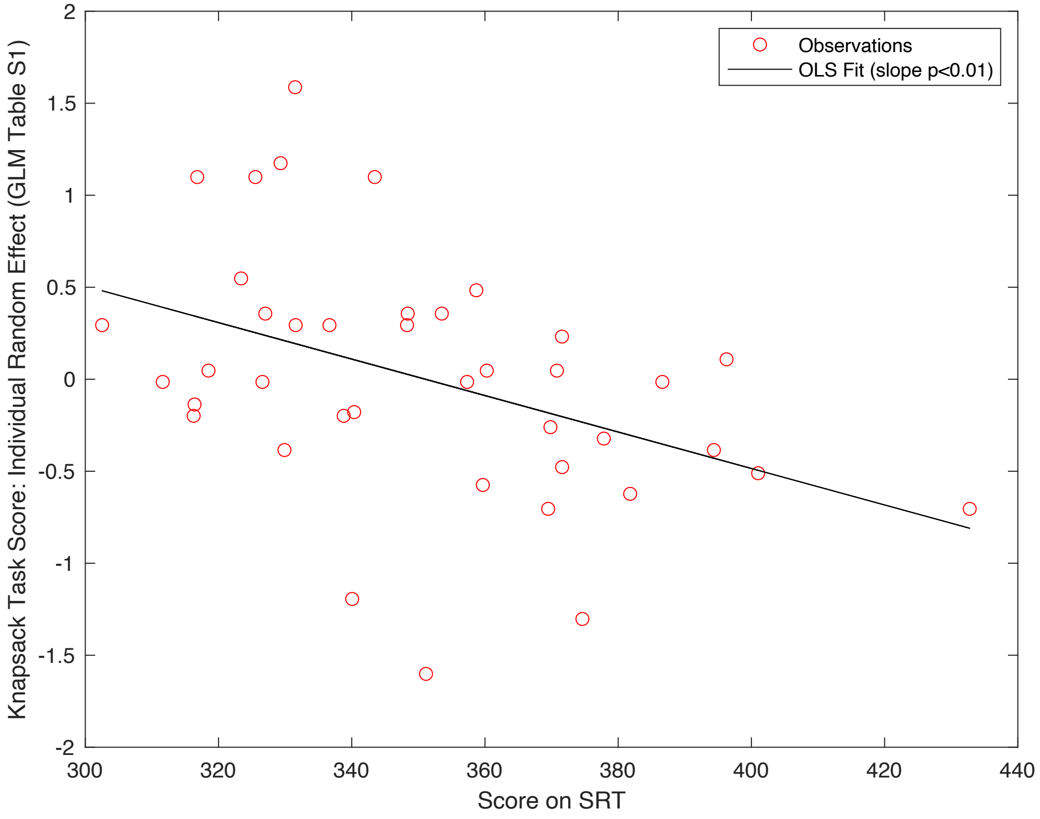

Fig. S9: Relation Average Score on Spatial Working Memory Task and Difference in Individual Deviation from Mean Effect on Percentage Correct Score under MOD vs. PLC Treatment

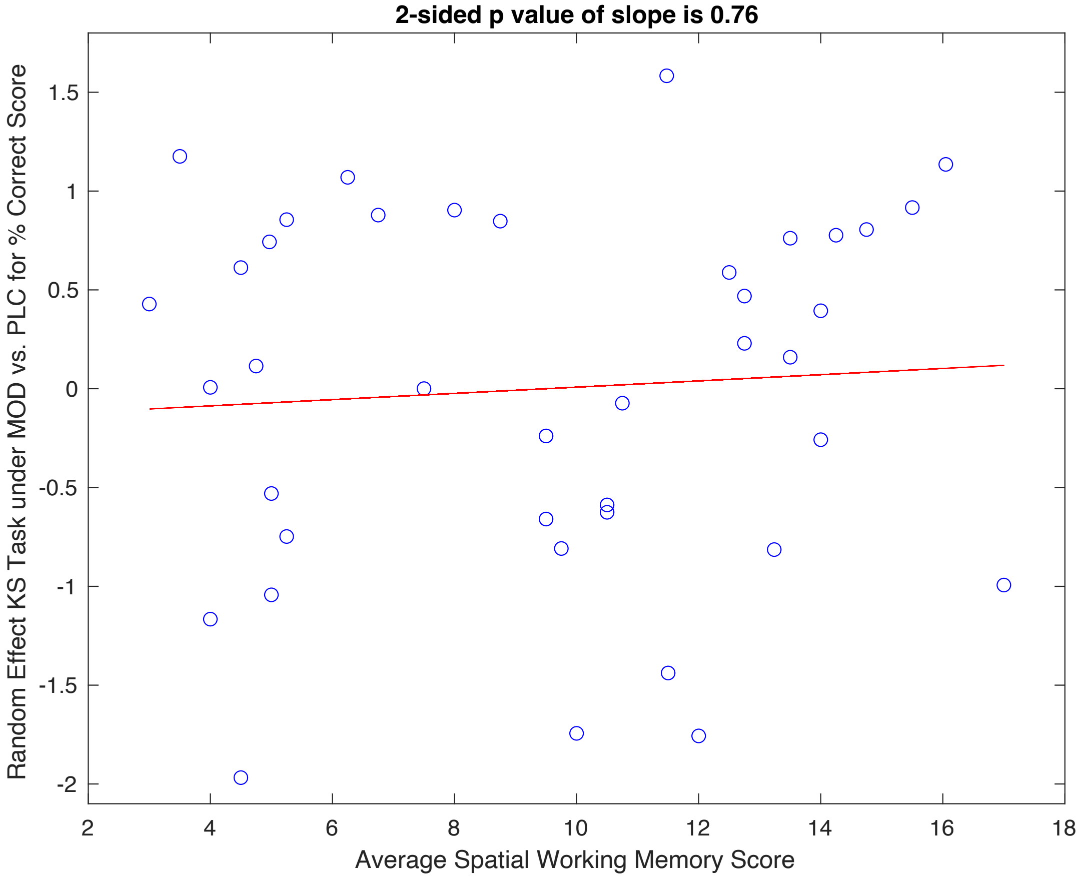

Fig. S10: Relation Average Score on Simple Reaction Time Task and Difference in Individual Deviation from Mean Effect on Percentage Correct Score under MOD vs. PLC Treatment

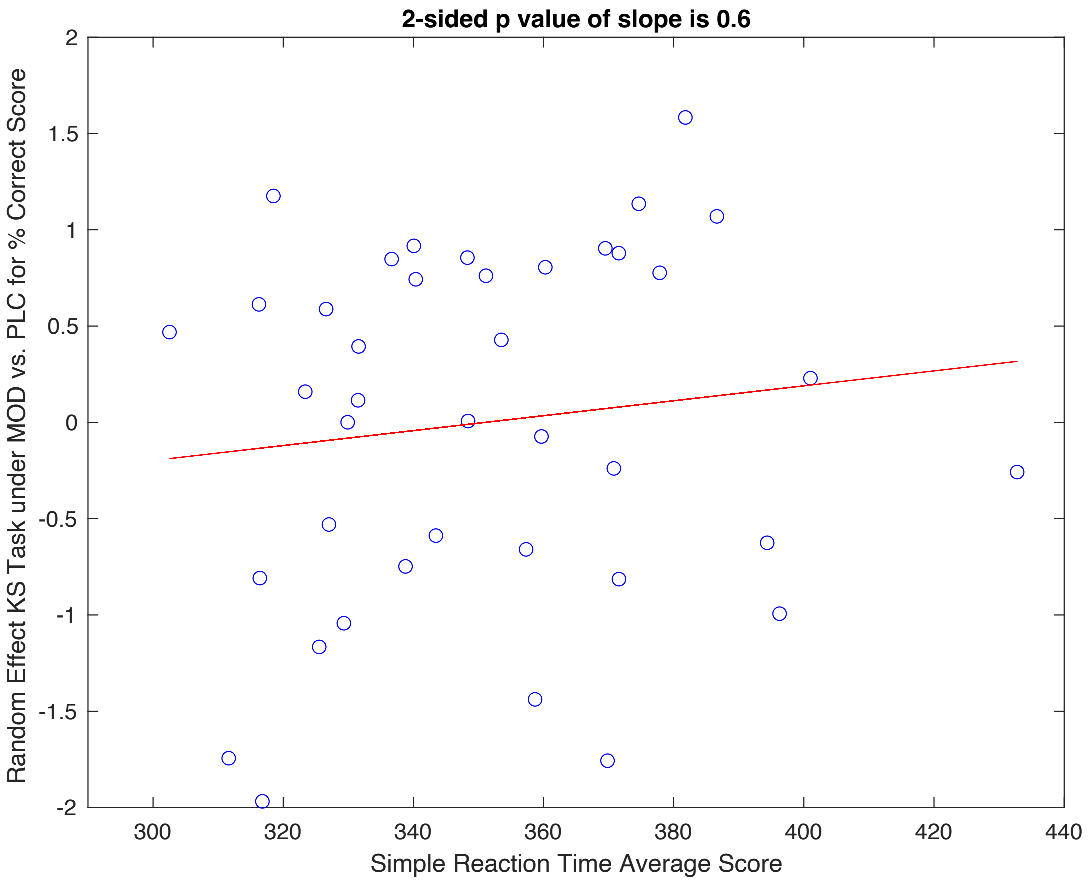

<u>Fig. S11</u>: Relation Average Score on Stockings Task and Difference in Individual Deviation from Mean Effect on Percentage Correct Score under MPH vs. PLC Treatment

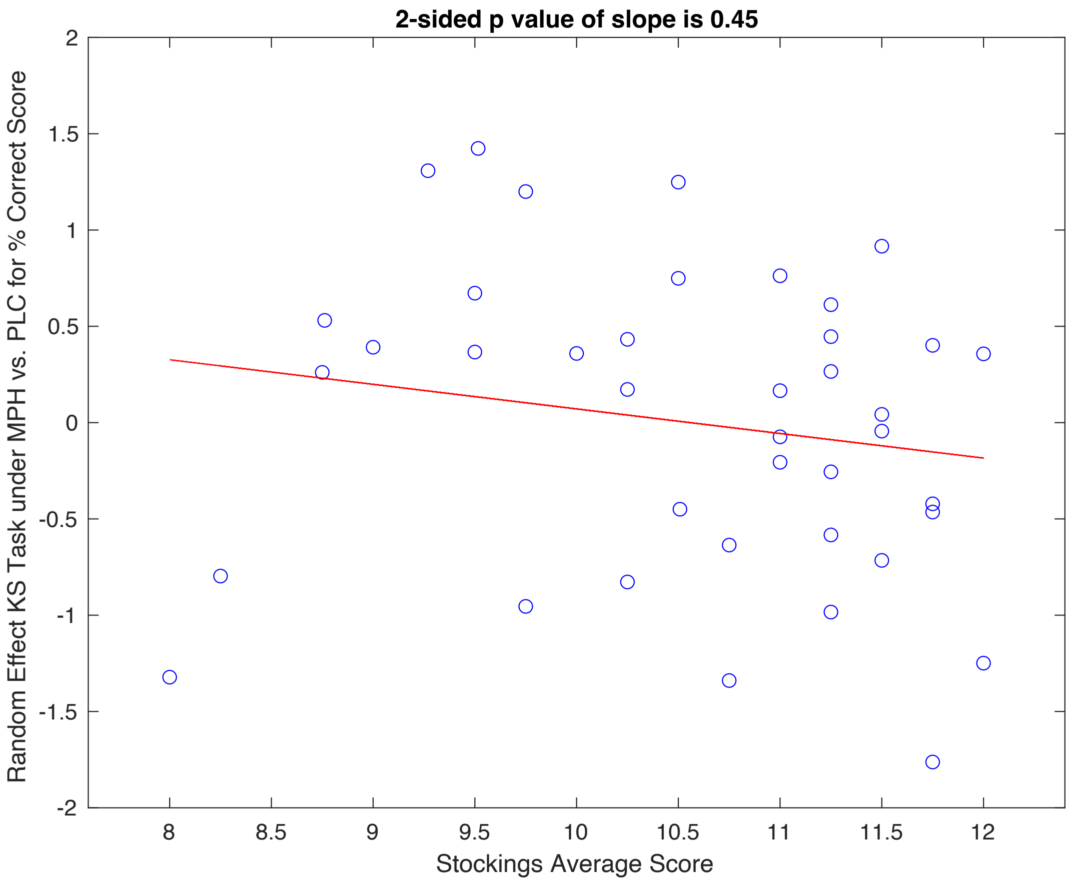

Fig. S12: Relation Average Score on Stop Signal Time Task and Difference in Individual Deviation from Mean Effect on Percentage Correct Score under DEX vs. PLC Treatment

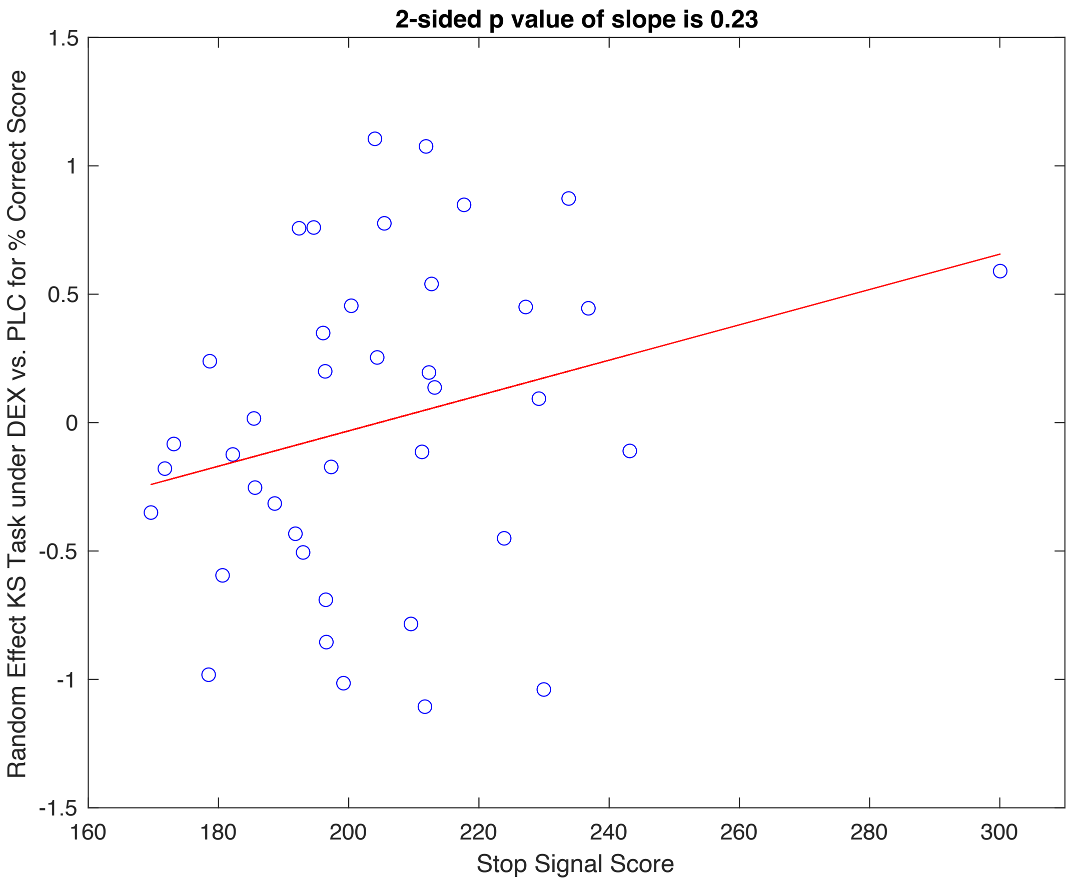

Fig. S13: Relation Drug Effect on Score on Spatial Working Memory Task (DEX vs. PLC) and Difference in Individual Deviation from Mean Effect on Percentage Correct Score under DEX vs. PLC Treatment

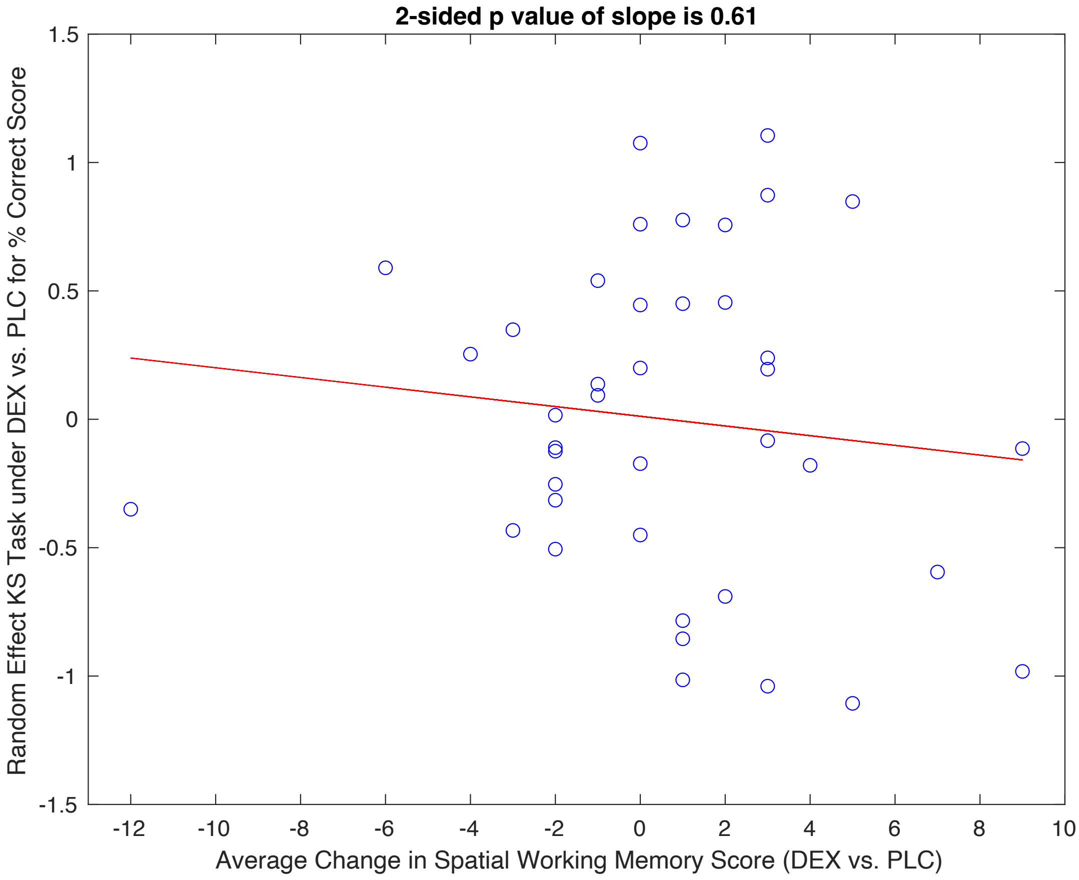

Fig. S14: Relation Drug Effect on Score on Simple Reaction Time Task (MPH vs. PLC) and Difference in Individual Deviation from Mean Effect on Percentage Correct Score under MPH vs. PLC Treatment

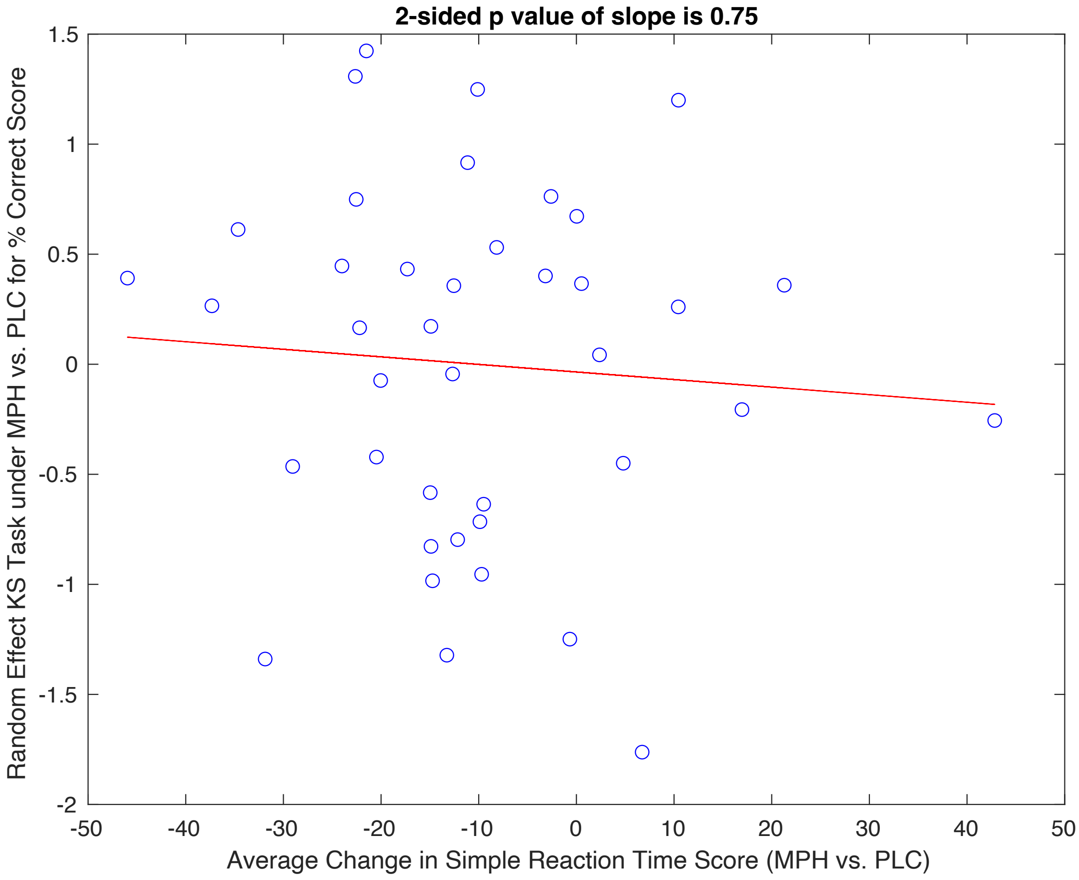

Fig. S15: Relation Drug Effect on Score on Stockings Task (MOD vs. PLC) and Difference in Individual Deviation from Mean Effect on Percentage Correct Score under MPH vs. PLC Treatment

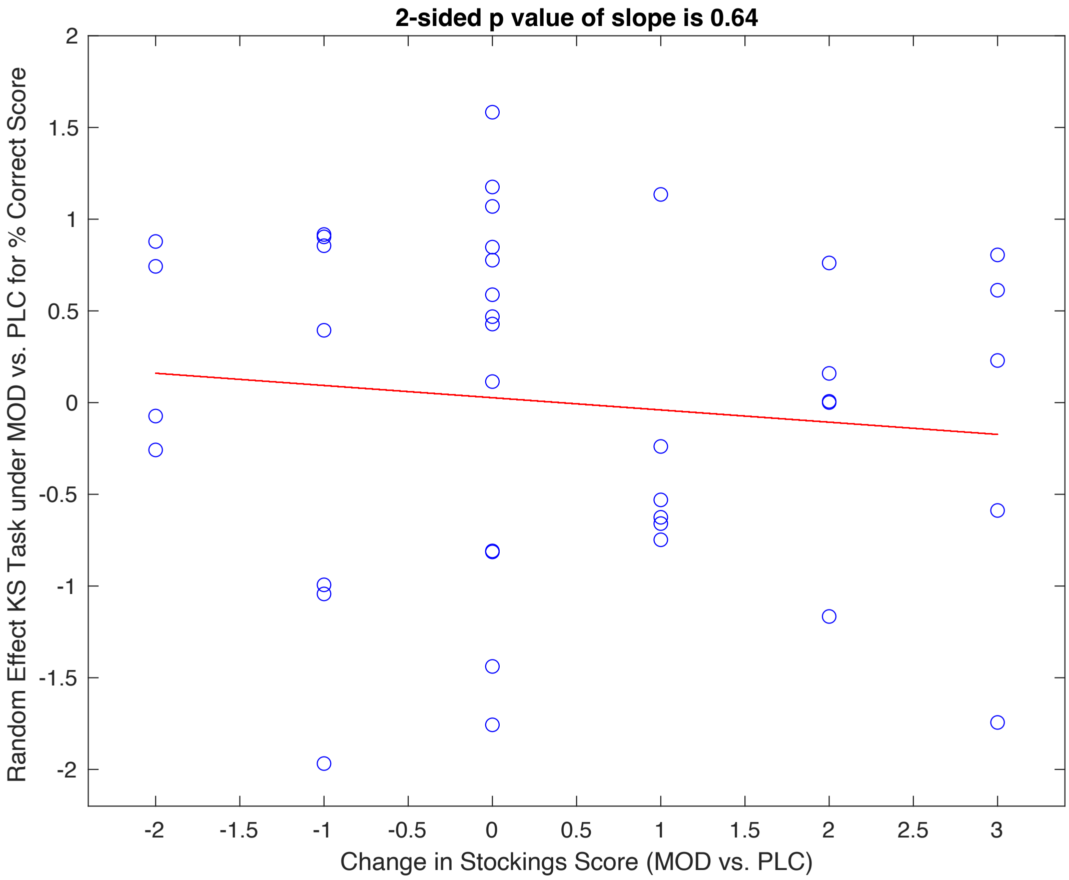

Fig. S16: Relation Drug Effect on Score on Stop Signal Time Task (DEX vs. PLC) and Difference in Individual Deviation from Mean Effect on Percentage Correct Score under DEX vs. PLC Treatment

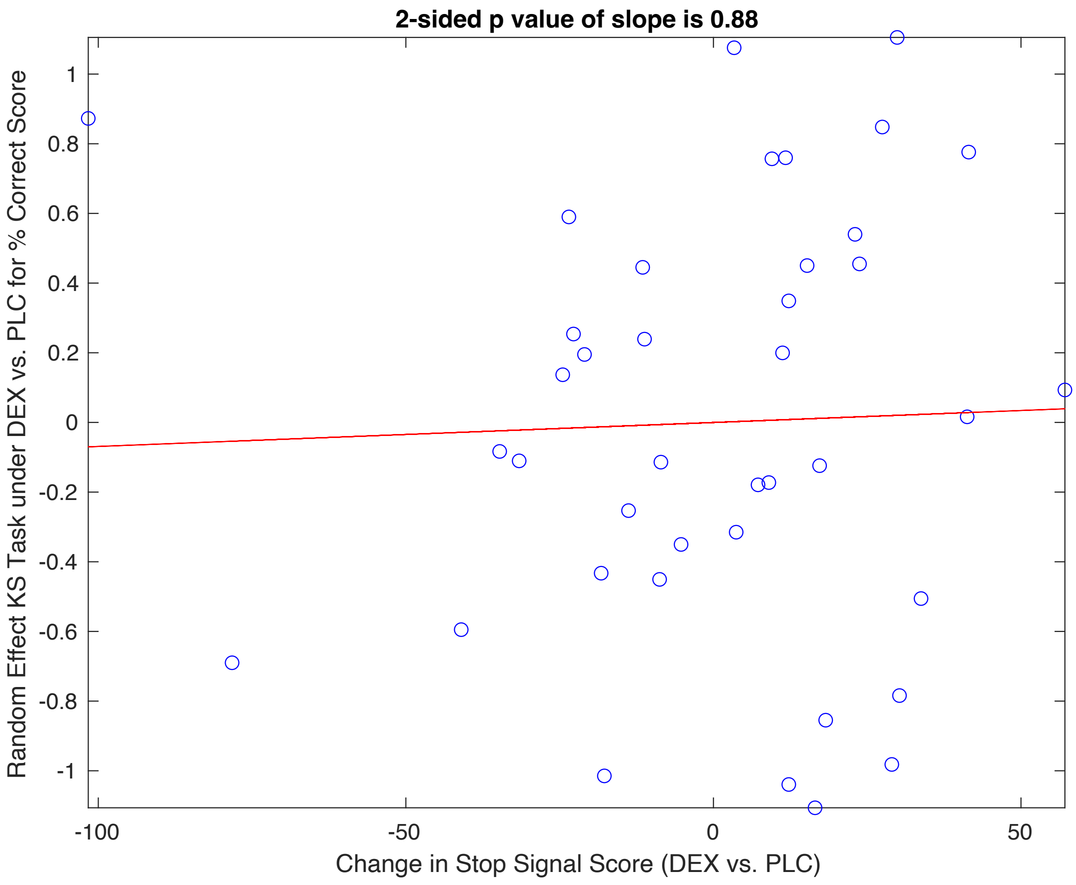

Supplementary Tables

**Notes:**

1. Matlab code that generates the tables, along with underlying data, can be found in the notebook "SOM.mlx" at https://zenodo.org/badge/latestdoi/592775835.
2. The Tables below contain output directly copied from the matlab command *fitglme*; this way the reader not only has a comprehensive list of statistics for each regression but also details of the regression specification, including where random effects are allowed for (*38*).

Table S1: GLM explaining score (submitted solution is correct (1)/incorrect (0))

Matlab commands:

```
modelspec = 'attempt_correct ~ sahni_k + Complexity + Drug + (1|participant_id)';
mdl = fitglme(Xall,modelspec,'Distribution','binomial','Link','logit')
```

*Note:* Complexity = $\log_2$(Knapsack Capacity)*(Number of Items)

Output:

```
mdl =
Generalized linear mixed-effects model fit by PL

Model information:
    Number of observations            2555
    Fixed effects coefficients           4
    Random effects coefficients         40
    Covariance parameters                1
    Distribution                  Binomial
    Link                             Logit
    FitMethod                          MPL

Formula:
    attempt_correct ~ 1 + sahni_k + Complexity + Drug + (1 | participant_id)

Model fit statistics:
    AIC      BIC      LogLikelihood    Deviance
    11352    11381    -5670.9          11342

Fixed effects coefficients (95% CIs):
    Name                Estimate      SE          tStat      DF      pValue        Lower         Upper
    {'(Intercept)'}     1.5811        0.22399     7.0591     2551    2.1518e-12     1.1419       2.0204
    {'sahni_k'    }    -0.53551       0.03598   -14.884      2551    4.1413e-48    -0.60606     -0.46496
    {'Complexity' }    -0.0043264     0.001572   -2.7522     2551    0.0059616     -0.0074089   -0.0012439
    {'Drug'       }    -0.16184       0.10032    -1.6133     2551    0.10681       -0.35856      0.034873

Random effects covariance parameters:
Group: participant_id (40 Levels)
    Name1              Name2              Type        Estimate
    {'(Intercept)'}    {'(Intercept)'}    {'std'}     0.70672

Group: Error
    Name                    Estimate
    {'sqrt(Dispersion)'}    1
```

Table S2: GLM explaining value attained as fraction of optimal value.

Matlab commands:

```
modelspec = 'attempt_value_pct_solution ~ sahni_k + Complexity + Drug + (1|participant_id)';
mdl = fitglme(Xall,modelspec,'Distribution','normal','Link','identity')
```

*Note:* mzn_props = number of propagations that the MiniZinc program took to find the solution of the instance.

Output:

```
mdl =
Generalized linear mixed-effects model fit by PL

Model information:
    Number of observations            2555
    Fixed effects coefficients           5
    Random effects coefficients         40
    Covariance parameters                2
    Distribution                    Normal
    Link                          Identity
    FitMethod                          MPL

Formula:
    attempt_value_pct_solution ~ 1 + sahni_k + mzn_props + Complexity + Drug + (1 | participant_id)

Model fit statistics:
    AIC       BIC       LogLikelihood    Deviance
    -10133    -10092    5073.5           -10147

Fixed effects coefficients (95% CIs):
    Name                 Estimate    SE            tStat     DF      pValue        Lower          Upper
    {'(Intercept)'}  0.96745      0.0034085   283.84     2550    0             0.96077        0.97414
    {'sahni_k'    }  0.0025469    0.00051941    4.9034   2550    1.001e-06     0.0015284      0.0035654
    {'mzn_props'  } -4.9492e-07   9.2191e-08   -5.3684   2550    8.6627e-08   -6.7569e-07    -3.1414e-07
    {'Complexity' }  0.0001751    2.735e-05     6.4022   2550    1.8167e-10    0.00012147     0.00022873
    {'Drug'       } -0.0034813    0.0014938    -2.3305   2550    0.019857     -0.0064106     -0.00055212

Random effects covariance parameters:
Group: participant_id (40 Levels)
    Name1                  Name2                  Type           Estimate
    {'(Intercept)'}        {'(Intercept)'}        {'std'}        0.011235

Group: Error
    Name                        Estimate
    {'sqrt(Dispersion)'}        0.032665
```

Table S3: GLM explaining time spent on instance

Matlab commands:

```
modelspec = 'time_on_task ~ sahni_k + Complexity + treatment_cat + (1 +
treatment_cat|participant_id)';
mdl = fitglme(Xall,modelspec,'Distribution','normal','Link','identity')
```

Output:

```
mdl =
Generalized linear mixed-effects model fit by PL

Model information:
    Number of observations            2555
    Fixed effects coefficients           7
    Random effects coefficients        160
    Covariance parameters               11
    Distribution                    Normal
    Link                          Identity
    FitMethod                          MPL

Formula:
    time_on_task ~ 1 + treatment_cat + sahni_k + mzn_props + Complexity + (1 + treatment_cat | participant_id)

Model fit statistics:
    AIC      BIC      LogLikelihood    Deviance
    27232    27337    -13598           27196

Fixed effects coefficients (95% CIs):
    Name                   Estimate      SE         tStat     DF     pValue        Lower      Upper
    {'(Intercept)'      }  69.627        7.28       9.5642    2548   2.565e-21    55.352      83.903
    {'treatment_cat_DEX'}  18.839        4.1608     4.5277    2548   6.2381e-06   10.68       26.997
    {'treatment_cat_MPH'}  29.074        4.8911     5.9443    2548   3.1571e-09   19.483      38.665
    {'treatment_cat_MOD'}   9.1276       5.4949     1.6611    2548   0.096813     -1.6472     19.902
    {'sahni_k'          }   6.9951       0.74555    9.3824    2548   1.3795e-20    5.5331      8.457
    {'mzn_props'        }   0.00039245   0.00013233 2.9657    2548   0.003048      0.00013297  0.00065194
    {'Complexity'       }   0.48031      0.039257  12.235     2548   1.7246e-33    0.40334     0.55729

Random effects covariance parameters:
Group: participant_id (40 Levels)
    Name1                       Name2                       Type              Estimate
    {'(Intercept)'      }       {'(Intercept)'      }       {'std' }          37.717
    {'treatment_cat_DEX'}       {'(Intercept)'      }       {'corr'}          -0.27607
    {'treatment_cat_MPH'}       {'(Intercept)'      }       {'corr'}          -0.482
    {'treatment_cat_MOD'}       {'(Intercept)'      }       {'corr'}          -0.52812
    {'treatment_cat_DEX'}       {'treatment_cat_DEX'}       {'std' }          20.416
    {'treatment_cat_MPH'}       {'treatment_cat_DEX'}       {'corr'}           0.19975
    {'treatment_cat_MOD'}       {'treatment_cat_DEX'}       {'corr'}           0.58317
    {'treatment_cat_MPH'}       {'treatment_cat_MPH'}       {'std' }          26.105
    {'treatment_cat_MOD'}       {'treatment_cat_MPH'}       {'corr'}           0.43541
    {'treatment_cat_MOD'}       {'treatment_cat_MOD'}       {'std' }          30.529

Group: Error
    Name                        Estimate
    {'sqrt(Dispersion)'}        46.886
```

Table S4: GLM explaining number of moves taken on instance

Matlab commands:

```
modelspec = 'length_of_sequence ~ sahni_k + Complexity + treatment_cat + (1 +
treatment_cat|participant_id)';
mdl = fitglme(Xall,modelspec,'Distribution','normal','Link','identity')
```

Output:

```
mdl =
Generalized linear mixed-effects model fit by PL

Model information:
    Number of observations            2555
    Fixed effects coefficients           7
    Random effects coefficients        160
    Covariance parameters               11
    Distribution                    Normal
    Link                            Identity
    FitMethod                       MPL

Formula:
    length_of_sequence ~ 1 + treatment_cat + sahni_k + mzn_props + Complexity + (1 + treatment_cat |
participant_id)

Model fit statistics:
    AIC      BIC      LogLikelihood    Deviance
    21159    21264    -10562           21123

Fixed effects coefficients (95% CIs):
    Name                      Estimate   SE          tStat     DF    pValue         Lower         Upper
    {'(Intercept)'      }   4.1251     1.9983      2.0643    2548  0.039091       0.20661       8.0435
    {'treatment_cat_DEX'}   7.2479     1.4295      5.0701    2548  4.2611e-07     4.4447       10.051
    {'treatment_cat_MPH'}   6.1308     1.51        4.0602    2548  5.0515e-05     3.1699        9.0917
    {'treatment_cat_MOD'}   1.941      1.5479      1.2539    2548  0.20998       -1.0943        4.9763
    {'sahni_k'          }   3.531      0.22757    15.516     2548  5.9449e-52     3.0848        3.9773
    {'mzn_props'        }  -0.00032061 4.0392e-05 -7.9374    2548  3.0646e-15    -0.00039981   -0.0002414
    {'Complexity'       }   0.17356    0.011983   14.484     2548  9.4943e-46     0.15007       0.19706

Random effects covariance parameters:
Group: participant_id (40 Levels)
    Name1                        Name2                        Type           Estimate
    {'(Intercept)'      }        {'(Intercept)'      }        {'std' }         9.7342
    {'treatment_cat_DEX'}        {'(Intercept)'      }        {'corr'}         0.34562
    {'treatment_cat_MPH'}        {'(Intercept)'      }        {'corr'}         0.16511
    {'treatment_cat_MOD'}        {'(Intercept)'      }        {'corr'}        -0.19622
    {'treatment_cat_DEX'}        {'treatment_cat_DEX'}        {'std' }         7.4871
    {'treatment_cat_MPH'}        {'treatment_cat_DEX'}        {'corr'}         0.52197
    {'treatment_cat_MOD'}        {'treatment_cat_DEX'}        {'corr'}         0.58633
    {'treatment_cat_MPH'}        {'treatment_cat_MPH'}        {'std' }         8.0955
    {'treatment_cat_MOD'}        {'treatment_cat_MPH'}        {'corr'}         0.58985
    {'treatment_cat_MOD'}        {'treatment_cat_MOD'}        {'std' }         8.376

Group: Error
    Name                       Estimate
    {'sqrt(Dispersion)'}       14.311
```

Table S5: GLM explaining speed (number of seconds per move)

Matlab commands:

```
modelspec = 'speed ~ sahni_k + Complexity + treatment_cat + (1 + treatment_cat|participant_id)';
mdl = fitglme(Xall,modelspec,'Distribution','normal','Link','identity')
```

Output:

```
mdl =
Generalized linear mixed-effects model fit by PL

Model information:
    Number of observations             2555
    Fixed effects coefficients            7
    Random effects coefficients         160
    Covariance parameters                11
    Distribution                     Normal
    Link                           Identity
    FitMethod                           MPL

Formula:
    speed ~ 1 + treatment_cat + sahni_k + mzn_props + Complexity + (1 + treatment_cat | participant_id)

Model fit statistics:
    AIC      BIC      LogLikelihood    Deviance
    14006    14112    -6985.2          13970

Fixed effects coefficients (95% CIs):
    Name                       Estimate    SE           tStat      DF      pValue        Lower        Upper
    {'(Intercept)'      }  9.7137      0.46524      20.879     2548    1.7054e-89   8.8014      10.626
    {'treatment_cat_DEX'} -0.36049     0.31839      -1.1322    2548    0.25764     -0.98482      0.26384
    {'treatment_cat_MPH'}  0.51183     0.30266       1.6911    2548    0.090944    -0.081663     1.1053
    {'treatment_cat_MOD'} -0.041949    0.31045      -0.13512   2548    0.89252     -0.6507       0.5668
    {'sahni_k'          } -0.5641      0.056582     -9.9696    2548    5.4311e-23  -0.67505     -0.45314
    {'mzn_props'        }  0.00010963  1.0043e-05   10.916     2548    3.8097e-27   8.9937e-05   0.00012932
    {'Complexity'       } -0.029372    0.0029793    -9.8585    2548    1.585e-22   -0.035214    -0.023529

Random effects covariance parameters:
Group: participant_id (40 Levels)
    Name1                        Name2                        Type            Estimate
    {'(Intercept)'      }        {'(Intercept)'      }        {'std' }          2.1543
    {'treatment_cat_DEX'}        {'(Intercept)'      }        {'corr'}          0.12189
    {'treatment_cat_MPH'}        {'(Intercept)'      }        {'corr'}          0.24351
    {'treatment_cat_MOD'}        {'(Intercept)'      }        {'corr'}         -0.28658
    {'treatment_cat_DEX'}        {'treatment_cat_DEX'}        {'std' }          1.5707
    {'treatment_cat_MPH'}        {'treatment_cat_DEX'}        {'corr'}          0.28239
    {'treatment_cat_MOD'}        {'treatment_cat_DEX'}        {'corr'}          0.57732
    {'treatment_cat_MPH'}        {'treatment_cat_MPH'}        {'std' }          1.4414
    {'treatment_cat_MOD'}        {'treatment_cat_MPH'}        {'corr'}          0.42524
    {'treatment_cat_MOD'}        {'treatment_cat_MOD'}        {'std' }          1.5057

Group: Error
    Name                        Estimate
    {'sqrt(Dispersion)'}        3.5583
```

Table S6: GLM explaining productivity (average gain in value per move as fraction of optimal value)

Matlab commands:

```
modelspec = 'productivity ~ 1 + sahni_k + Complexity + treatment_cat + (1 +
treatment_cat|participant_id)';
mdl = fitglme(Xall,modelspec,'Distribution','normal','Link','identity')
```

Output:

```
mdl =
Generalized linear mixed-effects model fit by PL

Model information:
    Number of observations            2555
    Fixed effects coefficients           7
    Random effects coefficients        160
    Covariance parameters               11
    Distribution                    Normal
    Link                          Identity
    FitMethod                          MPL

Formula:
    productivity ~ 1 + treatment_cat + sahni_k + mzn_props + Complexity + (1 + treatment_cat | participant_id)

Model fit statistics:
    AIC        BIC        LogLikelihood    Deviance
    -9279.6    -9174.4    4657.8           -9315.6

Fixed effects coefficients (95% CIs):
    Name                    Estimate    SE            tStat     DF      pValue        Lower        Upper
    {'(Intercept)'      }  0.12712     0.0049474    25.695    2548    1.1381e-129   0.11742      0.13683
    {'treatment_cat_DEX'} -0.012042    0.0031923    -3.7723   2548    0.00016543   -0.018302    -0.0057825
    {'treatment_cat_MPH'} -0.010754    0.0024325    -4.4209   2548    1.0239e-05   -0.015524    -0.005984
    {'treatment_cat_MOD'} -0.0072721   0.0034906    -2.0833   2548    0.03732      -0.014117    -0.00042738
    {'sahni_k'          } -0.0071917   0.00059731 -12.04     2548    1.637e-32    -0.0083629   -0.0060204
    {'mzn_props'        }  5.6351e-07  1.0602e-07   5.3153   2548    1.1574e-07    3.5562e-07   7.7139e-07
    {'Complexity'       } -0.00053636  3.1451e-05 -17.054    2548    7.784e-62    -0.00059803 -0.00047469

Random effects covariance parameters:
Group: participant_id (40 Levels)
    Name1                        Name2                        Type             Estimate
    {'(Intercept)'      }        {'(Intercept)'      }        {'std' }         0.023054
    {'treatment_cat_DEX'}        {'(Intercept)'      }        {'corr'}        -0.21123
    {'treatment_cat_MPH'}        {'(Intercept)'      }        {'corr'}        -0.425
    {'treatment_cat_MOD'}        {'(Intercept)'      }        {'corr'}        -0.55189
    {'treatment_cat_DEX'}        {'treatment_cat_DEX'}        {'std' }         0.015188
    {'treatment_cat_MPH'}        {'treatment_cat_DEX'}        {'corr'}        -0.49508
    {'treatment_cat_MOD'}        {'treatment_cat_DEX'}        {'corr'}         0.69938
    {'treatment_cat_MPH'}        {'treatment_cat_MPH'}        {'std' }         0.0077381
    {'treatment_cat_MOD'}        {'treatment_cat_MPH'}        {'corr'}         0.041278
    {'treatment_cat_MOD'}        {'treatment_cat_MOD'}        {'std' }         0.017619

Group: Error
    Name                        Estimate
    {'sqrt(Dispersion)'}        0.037563
```

Table S7: GLM explaining quality of first full knapsack attempted (measured as number of items common to the first full knapsack and the optimal knapsack)

Matlab commands:

```
modelspec = 'num_items_overlap_first_tn_sol_set ~ 1 + sahni_k + Drug +
sahni_k*num_items_overlap_GA_sol + (1|participant_id)';
mdl = fitglme(Xall,modelspec,'Distribution','normal','Link','identity')
```

*Note:* `num_items_overlap_GA_sol = number of items common to the solution from the Greedy Algorithm and the optimal knapsack`

Output:

```
mdl =
Generalized linear mixed-effects model fit by PL

Model information:
    Number of observations            2555
    Fixed effects coefficients           5
    Random effects coefficients         40
    Covariance parameters                2
    Distribution                    Normal
    Link                          Identity
    FitMethod                          MPL

Formula:
    num_items_overlap_first_tn_sol_set ~ 1 + Drug + sahni_k*num_items_overlap_GA_sol + (1 | participant_id)

Model fit statistics:
    AIC     BIC       LogLikelihood    Deviance
    8602    8642.9    -4294            8588

Fixed effects coefficients (95% CIs):
    Name                                  Estimate SE        tStat    DF    pValue       Lower    Upper
    {'(Intercept)'                      } 2.053    0.13819   14.856   2550  6.0347e-48   1.7821   2.324
    {'sahni_k'                          }-1.1946   0.06521  -18.32    2550  1.5825e-70  -1.3225  -1.0668
    {'num_items_overlap_GA_sol'         } 0.49242  0.019981  24.645   2550  1.8054e-120  0.45324  0.5316
    {'Drug'                             }-0.17583  0.058579  -3.0017  2550  0.0027111   -0.2907  -0.060967
    {'sahni_k:num_items_overlap_GA_sol'} 0.33602  0.012548  26.78    2550  1.9812e-139  0.31142  0.36063

Random effects covariance parameters:
Group: participant_id (40 Levels)
    Name1                  Name2                  Type           Estimate
    {'(Intercept)'}        {'(Intercept)'}        {'std'}        0.36005

Group: Error
    Name                       Estimate
    {'sqrt(Dispersion)'}       1.2809
```

## REFERENCES AND NOTES


1. E. Bowman, B. Feng, C. Murawski, P. Bossaerts, *Surveying the Use of Pharmaceutical Cognitive Enhancers in the Australian Financial Services Industry* (SSRN Scholarly Paper 3661966, Social Science Research Network, 2020).

2. P. Dietz, M. Soyka, A. G. Franke, Pharmacological neuroenhancement in the field of economics—Poll results from an online survey. *Front. Psychol.* **7**, 520 (2016).

3. R. M. Emanuel, S. L. Frellsen, K. J. Kashima, S. M. Sanguino, F. S. Sierles, C. J. Lazarus, Cognitive enhancement drug use among future physicians: Findings from a multi-institutional census of medical students. *J. Gen. Intern. Med.* **28**, 1028–1034 (2013).

4. A. F. Kortekaas-Rijlaarsdam, M. Luman, E. Sonuga-Barke, J. Oosterlaan, Does methylphenidate improve academic performance? A systematic review and meta-analysis. *Eur. Child Adolesc. Psychiatry* **28**, 155–164 (2019).

5. I. Ilieva, J. Boland, M. J. Farah, Objective and subjective cognitive enhancing effects of mixed amphetamine salts in healthy people. *Neuropharmacology* **64**, 496–505 (2013).

6. S. R. Chamberlain, T. W. Robbins, S. Winder-Rhodes, U. Müller, B. J. Sahakian, A. D. Blackwell, J. H. Barnett, Translational approaches to frontostriatal dysfunction in attention-deficit/hyperactivity disorder using a computerized neuropsychological battery. *Biol. Psychiatry* **69**, 1192–1203 (2011).

7. D. Repantis, P. Schlattmann, O. Laisney, I. Heuser, Modafinil and methylphenidate for neuroenhancement in healthy individuals: A systematic review. *Pharmacol. Res.* **62**, 187–206 (2010).

8. M. E. Smith, M. J. Farah, Are prescription stimulants “smart pills”? The epidemiology and cognitive neuroscience of prescription stimulant use by normal healthy individuals *Psychol. Bull.* **137**, 717–741 (2011).

9. R. M. Battleday, A.-K. Brem, Modafinil for cognitive neuroenhancement in healthy non-sleep-deprived subjects: A systematic review. *Eur. Neuropsychopharmacol.* **25**, 1865–1881 (2015).

10. D. R. Coghill, S. M. Rhodes, K. Matthews, The neuropsychological effects of chronic methylphenidate on drug-naive boys with attention-deficit/hyperactivity disorder. *Biol. Psychiatry* **62**, 954–962 (2007).

11. R. H. Pietrzak, C. M. Mollica, P. Maruff, P. J. Snyder, Cognitive effects of immediate-release methylphenidate in children with attention-deficit/hyperactivity disorder. *Neurosci. Biobehav. Rev.* **30**, 1225–1245 (2006).

12. S. Arora, B. Barak, *Computational Complexity: A Modern Approach* (Cambridge Univ. Press, 2009).

13. N. Yadav, C. Murawski, S. Sardina, P. Bossaerts. Is hardness inherent in computational problems? Performance of human and electronic computers on random instances of the 0-1 knapsack problem, in *ECAI 2020* (IOS Press, 2020), pp. 498–505.

14. T. E. Wilens, Mechanism of action of agents used in attention-deficit/hyperactivity disorder. *J. Clin. Psychiatry* **67**, 11408 (2006).

15. R. Kuczenski, D. S. Segal, Effects of methylphenidate on extracellular dopamine, serotonin, and norepinephrine: Comparison with amphetamine. *J. Neurochem.* **68**, 2032–2037 (1997).

16. E. Mignot, S. Nishino, C. Guilleminault, W. Dement, Modafinil binds to the dopamine uptake carrier site with low affinity. *Sleep* **17**, 436–7 (1994).

17. D. Zolkowska, R. Jain, R. B. Rothman, J. S. Partilla, B. L. Roth, V. Setola, T. E. Prisinzano, M. H. Baumann, Evidence for the involvement of dopamine transporters in behavioral stimulant effects of modafinil. *J. Pharmacol. Exp. Ther.* **329**, 738–746 (2009).

18. B. K. Madras, Z. Xie, Z. Lin, A. Jassen, H. Panas, L. Lynch, R. Johnson, E. Livni, T. J. Spencer, A. A. Bonab, G. M. Miller, A. J. Fischman, Modafinil occupies dopamine and norepinephrine transporters in vivo and modulates the transporters and trace amine activity in vitro. *J. Pharmacol. Exp. Ther.* **319**, 561–569 (2006).

19. L. Ferraro, T. Antonelli, W. T. O'Connor, S. Tanganelli, F. Rambert, K. Fuxe, The antinarcoleptic drug modafinil increases glutamate release in thalamic areas and hippocampus. *Neuroreport* **8**, 2883–2887 (1997).

20. L. Ferraro, S. Tanganelli, W. T. O’Connor, T. Antonelli, F. Rambert, K. Fuxe, The vigilance promoting drug modafinil decreases GABA release in the medial preoptic area and in the posterior hypothalamus of the awake rat: Possible involvement of the serotonergic 5-HT3 receptor. *Neurosci. Lett.* **220**, 5–8 (1996).

21. CANTAB [Cognitive assessment software] (Cambridge Cognition, 2019); www.cantab.com.

22. D. Meloso, J. Copic, P. Bossaerts, Promoting intellectual discovery: Patents versus markets. *Science* **323**, 1335–1339 (2009).

23. C. Murawski, P. Bossaerts, How humans solve complex problems: The case of the knapsack problem. *Sci. Rep.* **6**, 34851 (2016).

24. J. P. Franco, N. Yadav, P. Bossaerts, C. Murawski, Generic properties of a computational task predict human effort and performance. *J. Math. Psychol.* **104**, 102592 (2021).

25. D. Pisinger, P. Toth, Knapsack problems, in *Handbook of Combinatorial Optimization: Volume1–3*, D.-Z. Du, P. M. Pardalos, Eds. (Springer, 1998); pp. 299–428; https://doi.org/10.1007/978-1-4613-0303-9_5.

26. N. Nethercote, P. J. Stuckey, R. Becket, S. Brand, G. J. Duck, G. Tack, MiniZinc: Towards a standard CP modelling language, in *Principles and Practice of Constraint Programming—CP 2007*, C. Bessière, Ed. (Lecture Notes in Computer Science, Springer, 2007), pp. 529–543.

27. P. L. Yudkin, I. M. Stratton, How to deal with regression to the mean in intervention studies. *Lancet* **347**, 241–243 (1996).

28. R. S. Sutton, A. G. Barto, *Reinforcement Learning: An Introduction* (MIT Press, ed. 2, 2018).

29. N. Agay, E. Yechiam, Z. Carmel, Y. Levkovitz, Non-specific effects of methylphenidate (Ritalin) on cognitive ability and decision-making of ADHD and healthy adults. *Psychopharmacology* **210**, 511–519 (2010).

30. N. Agay, E. Yechiam, Z. Carmel, Y. Levkovitz, Methylphenidate enhances cognitive performance in adults with poor baseline capacities regardless of attention-deficit/hyperactivity disorder diagnosis. *J. Clin. Psychopharmacol.* **34**, 261–265 (2014).

31. D. Campbell-Meiklejohn, A. Simonsen, J. Scheel-Krüger, V. Wohlert, T. Gjerløff, C. D. Frith, R. D. Rogers, A. Roepstorff, A. Møller, In for a penny, in for a pound: Methylphenidate reduces the inhibitory effect of high stakes on persistent risky choice. *J. Neurosci.* **32**, 13032–13038 (2012).

32. S. R. Schroeder, K. Mann-Koepke, C. T. Gualtieri, D. A. Eckerman, G. R. Breese, Methylphenidate affects strategic choice behavior in normal adult humans. *Pharmacol. Biochem. Behav.* **28**, 213–217 (1987).

33. C. Bellebaum, L. Kuchinke, P. Roser, Modafinil alters decision making based on feedback history—A randomized placebo-controlled double blind study in humans. *J. Psychopharmacol.* **31**, 243–249 (2017).

34. D. C. Turner, T. W. Robbins, L. Clark, A. R. Aron, J. Dowson, B. J. Sahakian, Cognitive enhancing effects of modafinil in healthy volunteers. *Psychopharmacology* **165**, 260–269 (2003).

35. M. E. Walton, S. Bouret, What is the relationship between dopamine and effort? *Trends Neurosci.* **42**, 79–91 (2019).

36. J. M. Swanson, T. L. Wigal, N. D. Volkow, Contrast of medical and nonmedical use of stimulant drugs, basis for the distinction, and risk of addiction: Comment on Smith and Farah (2011). *Psychol. Bull.* **137**, 742–748 (2011).

37. I. M. E. Idema, J. M. Payne, D. Coghill, Effects of methylphenidate on cognitive functions in boys with attention deficit hyperactivity disorder: Does baseline performance matter? *J. Consult. Clin. Psychol.* **89**, 615–625 (2021).

38. C. E. Rogers, Algorithm AS 65: Interpreting structure formulae *Appl. Stat.* **22**, 414–424 (1973).
